# Enhanced Selectivity in 4-Quinolone Formation: A Dual-Base System for Palladium-Catalyzed Carbonylative Cyclization with Fe(CO)_5_

**DOI:** 10.3390/molecules29040850

**Published:** 2024-02-14

**Authors:** Meng Guo, Dou Wu, Hongyu Yang, Xiao Zhang, Dong-Xu Xue, Weiqiang Zhang

**Affiliations:** 1 Key Laboratory of Applied Surface and Colloid Chemistry (MOE), School of Chemistry and Chemical Engineering, Shaanxi Normal University, Xi’an 710119, China; 2 Xi’an Key Laboratory of Organometallic Material Chemistry, School of Chemistry and Chemical Engineering, Shaanxi Normal University, Xi’an 710119, China

**Keywords:** 4-quinolone, Pd catalysis, carbonylation, CO-releasing molecule, mechanism

## Abstract

The use of gaseous CO in Pd-catalyzed carbonylative quinolone synthesis presents challenges related to safety and precise pressure control. In response, a streamlined non-gaseous synthesis of 4-quinolone compounds has been developed. This study introduces a tunable CO-releasing system utilizing Fe(CO)_5_ activated by a dual-base system of piperazine and triethylamine. This alternative liquid CO resource facilitates the palladium-catalyzed carbonylative C-C coupling and subsequent intramolecular cyclization. By tuning the tandem kinetics of carbonylation and cyclization, this non-gaseous method achieves the successful synthesis of 22 distinct 4-quinolones with excellent yields. This is achieved through the three-component condensation of sub-stoichiometric amounts of Fe(CO)_5_ with 2-iodoaniline and terminal alkynes. Operando mechanistic studies have revealed a novel CO transfer mechanism that facilitates homogeneous carbonylative cyclization, distinguishing this method from traditional techniques. In addition to addressing safety concerns, this approach also provides precise control over selectivity, with significant implications for pharmaceutical research and the efficient synthesis of pharmaceutical and bioactive compounds.

## 1. Introduction

**Scheme 1:**
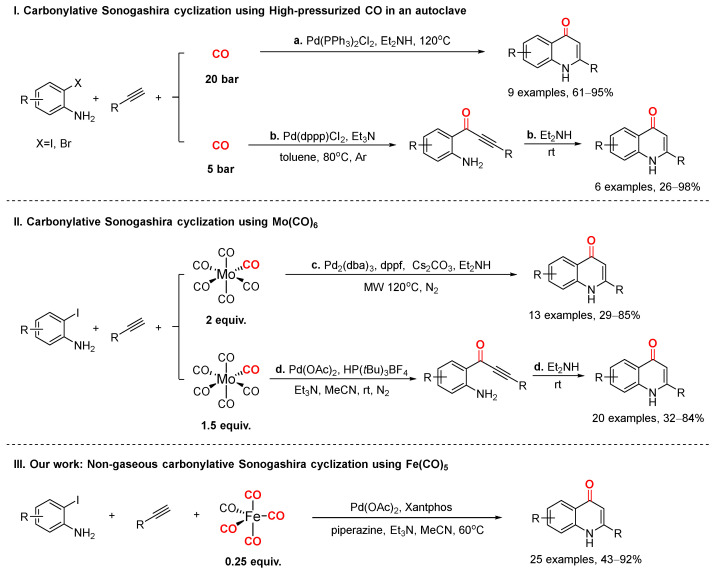
Palladium-catalyzed carbonylative Sonogashira cyclization for synthesizing 4-quinolones.

Quinolones are nitrogen-containing heterocyclic compounds that are widely found in natural products, pharmaceuticals, and biologically active molecules [1,2]. The palladium-catalyzed three-component carbonylative cyclization reaction is a highly regarded and efficient method for synthesizing quinolones [3,4]. The seminal work by Torii et al. in 1991 laid the foundation for this methodology (Scheme 1(I-a)) [5]. Subsequent research by Haddad et al. [6] and Djakovitch’s group (Scheme 1(I-b)) [7] further optimized the carbonylative cyclization by reducing CO pressure while achieving impressive yields. Ongoing efforts to enhance catalytic efficiency and sustainability include the use of selected palladium catalysts such as Pd(dppf)Cl_2_ (dppf = 1,1′-bis(diphenylphosphino)ferrocene) [6,8], Pd(dppp)Cl_2_ (dppp = 1,3-bis(diphenylphosphino)propane) [7], and [PdPNP]@SBA-15/[N]@SBA-3 [9]. Furthermore, Lei et al. developed a palladium/copper bimetallic-catalyzed three-component oxidative carbonylation synthesis of 4-quinolones under mild CO/O_2_ conditions, highlighting the evolving frontier of carbonylative cyclization [10]. These studies collectively underscore the pivotal role of CO as a versatile C1 source for generating carbonyl-containing compounds, emphasizing the continued relevance and potential of this synthetic strategy in heterocyclic chemistry.

Despite these advancements, the use of CO gas presents limitations such as safety concerns and handling requirements, necessitating the exploration of alternative and sustainable carbonylation methods [11]. To address the challenges associated with the use of toxic CO gas, researchers have investigated the use of Mo(CO)_6_ as carbon monoxide-releasing molecules (CORMs) for the non-gaseous synthesis of 4-quinolones. Larhed et al. demonstrated promising results by utilizing Mo(CO)_6_ as a solid CORM in both one-pot and stepwise palladium-catalyzed Sonogashira carbonylative cyclization reactions (Scheme 1(II)) [12]. Their one-pot synthesis, facilitated by microwave irradiation, showcased the efficient release of CO from two equivalents of Mo(CO)_6_, enabling its subsequent insertion into the 4-quinolone product (Scheme 1(II-c)). Additionally, Das et al. [13] and Wu’s group [14] demonstrated the versatility of CORMs in diverse reaction conditions by utilizing Pd-NHC and Pd/g-C_3_N_4_ as catalysts, respectively. However, successful non-gaseous carbonylative cyclization reactions require precise control over the kinetics of palladium-catalyzed carbonylation and intramolecular cyclization. The potential safety concerns associated with high temperature and pressure further emphasize the need for the development of efficient CO-releasing strategies under mild reaction conditions, highlighting the pressing need for the practical implementation of non-gaseous carbonylative cyclization.

Fe(CO)_5_ [15,16], known as iron pentacarbonyl, exhibits controlled and highly adaptable CO-releasing capabilities, offering several advantages such as safe syringe able handling, non-toxic byproducts, and a high CO utility rate. These qualities make it a versatile source of CO for various catalytic reactions, providing a convenient and efficient means of introducing CO into catalytic processes. Our group, along with others, has successfully used Fe(CO)_5_ as a liquid CORM for synthesizing carbonyl-containing compounds via various palladium-catalyzed carbonylative cross-coupling reactions, including Suzuki–Miyaura [17], Heck [18], and C-X (X = O and N) [19] coupling reactions.

Building upon the controlled and highly adaptable CO-releasing potential of Fe(CO)_5_, our hypothesis focuses on modulating the kinetic balance between palladium-catalyzed carbonylative coupling and cyclization in tandem to significantly improve the efficiency of three-component 4-quinolone synthesis. In this study, we present a highly selective Pd-catalyzed carbonylative cyclization method regulated by a dual-base modulated Fe(CO)_5_ as a CORM (Scheme 1(III)). This approach overcomes the limitations associated with traditional methods and offers a practical and efficient solution for the synthesis of 4-quinolones under mild conditions without the need for gaseous reagents.

## 2. Results and Discussion

Fe(CO)_5_, a stable liquid compound, offers several advantages over gaseous CO, simplifying handling, storage, and manipulation without requiring specialized high-pressure equipment or inert atmosphere protection. By using Fe(CO)_5_ as a liquid carbonyl source in a three-component carbonylation reaction, we investigated various reaction parameters and gained valuable insights (Table 1). The selectivity comparison of 4-quinolone (**4a**), uncyclized ynone (**5a**), and uncarbonylated alkyne (**6a**) revealed the significant impact of the base on the reaction’s selectivity. Notably, the presence of piperazine, potassium carbonate, and potassium phosphate resulted in moderate to low yields of **4a** with a substantial amount of **6a**, while **5a** was not produced (entries 1–3). Conversely, the use of triethylamine or TMEDA resulted in a small amount of **5a** and a significant reduction in **6a** yield (entries 4 and 5). The role of the base is demonstrated by the distribution of the by-products, indicating the significant effect of triethylamine on the carbonylative coupling and the advantageous role of piperazine in intramolecular cyclization.

The yields of 57% and 53% were obtained using piperazine and triethylamine, respectively, as individual bases in the reaction (entries 1 and 4). However, when these two bases were added together to the reaction system, the yield of **4a** increased to 70% with 3 equiv. piperazine and 1 equiv. triethylamine (entry 6). Furthermore, 1 equiv. piperazine resulted in complete conversion of the uncyclized product **5a**, 3 equiv. triethylamine significantly increased the substrate conversion rate of uncarbonylated product **6a**, leading to an 85% yield for **4a** (entry 7). These observations highlight the synergistic effect of the dual-base system in achieving optimal outcomes in tandem with the palladium-catalyzed carbonylation and cyclization reactions.

The dual-base system of triethylamine and piperazine was evaluated with various pre-catalysts and ligands (entries 7–11). In the absence of ligands, the substrates were not fully converted, and bivalent Pd(OAc)_2_ provided a higher yield of **4a** than the zero-valent Pd_2_(dba)_3_ (entries 7 and 8). Adding phosphine ligands significantly facilitated the substrate conversion, and the yields of the target product **4a** depended on the type of ligands (entries 9–11). Monodentate triphenylphosphine caused a decreased yield of **4a** to 66%, while the bidentate dppp and 9,9-dimethyl-4,5-bis(diphenylphosphino)xanthene (Xantphos) both improved the yield of **4a** compared with the ligand-free condition. The combination of Pd(OAc)_2_ and Xantphos exhibited excellent reactive efficiency, affording **4a** in 97% yield (entry 11). Even when the amount of Fe(CO)_5_ was reduced to half (0.25 equiv.), **4a** was still obtained with 90% yield (entry 12). Further reducing the amount of Fe(CO)_5_ to 0.2 equiv., the yield of **4a** dropped to 78%. Therefore, considering the carbonyl utilization efficiency in this synthetic method, 0.25 equiv. Fe(CO)_5_ was identified as the optimal amount of carbonyl source.

**Scheme 2:**
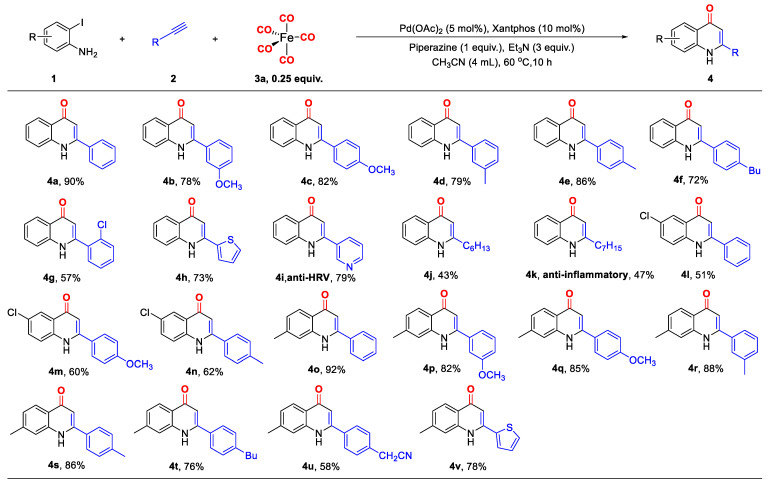
Substrate scope of 2-iodoanilines and terminal alkynes. Reaction conditions: 2-iodoaniline (0.5 mmol), Alkynyl reagent (1.2 equiv.), Pd(OAc)_2_ (5 mol%), Xantphos (10 mol%), Piperazine (1.0 equiv.), Et_3_N (3.0 equiv.), Fe(CO)_5_ (0.25 equiv.), and CH_3_CN (4 mL) was stirred at 60 °C for 10 h. Isolated yield.

After establishing non-gaseous catalytic carbonylation conditions, we investigated the substrate scope and limitations of the reaction, exploring a diverse range of terminal alkynes and 2-iodoanilines (Scheme 2). In the carbonylative Sonogashira cyclization reaction, we observed that nonfunctionalized phenylacetylene yielded the corresponding 4-quinolones (**4a**) with excellent yields, surpassing phenylacetylenes with methoxyl groups at para- and meta-positions (**4b**, **4c**). Notably, phenylacetylenes bearing methyl groups at the para-position exhibited higher reactivity than those at the meta-position (**4d**, **4e**), with similar trends observed for methoxyl groups at these positions. However, when extending the substituent carbon chain to n-butyl, the corresponding product **4f** yielded lower than **4e**. Additionally, phenylacetylenes with electron-withdrawing substituents such as chlorine demonstrated moderate yields (**4g**).

Furthermore, we successfully obtained heteroatom-containing thienylquinolone **4h** and pyridinoquinolone **4i** in good yields. Notably, **4i** is a well-known non-covalent inhibitor of the 3C proteinase of the coronavirus [20]. The linear alkyl-substituted products **4j** and **4k** were obtained in 43% and 47% yields, with **4k** showing potential in ameliorating a lipopolysaccharide-induced inflammatory response [21]. Compared to aromatic alkynes, aliphatic alkynes exhibited slightly lower reactivity. Subsequently, chloro-substituted 2-iodoaniline separately reacted with phenylacetylenes bearing hydrogen, methoxy, and methyl groups, yielding the corresponding products (**4l**–**4n**) in moderate yields. It is worth noting that the introduction of an electron-donating methyl group in 2-iodoaniline exhibited higher reactivity compared to the unsubstituted 2-iodoaniline, resulting in products **4o**–**4v**.

The practical selection of carbonyl sources is crucial for achieving efficient palladium-catalyzed three-component carbonylative cyclization reactions. The synthetic conditions required for various carbonyl sources as reported in the literature were listed in Table 2. The yield of unsubstituted product **4a** reflects the synthetic efficiency of different methods. Under a 20 bar CO gas and high temperature condition, **4a** was synthesized with a high yield of 90% (entry 1). At 5 bar CO atmosphere, the carbonylation and cyclization reactions were carried out in a stepwise manner with a significant decrease in the yield of **4a**. 2 equiv. (entry 2). Mo(CO)_6_ as a CORM afforded **4a** with an 85% yield at microwave 120 °C (entry 3). In a stepwise synthesis approach, the amount of Mo(CO)_6_ was reduced to 1.5 equiv., and 84% yield of 4a was obtained at room temperature (entry 4). Significantly, only 0.25 equiv. Fe(CO)_5_ was used as a CORM at 60 °C generated **4a** in 91% yield (entry 5). The mild conditions for CO release and outstanding carbonyl utilization in palladium catalyzed carbonylative cyclization resulted in the efficient synthesis of 4-quinolones.

**Figure 1:**
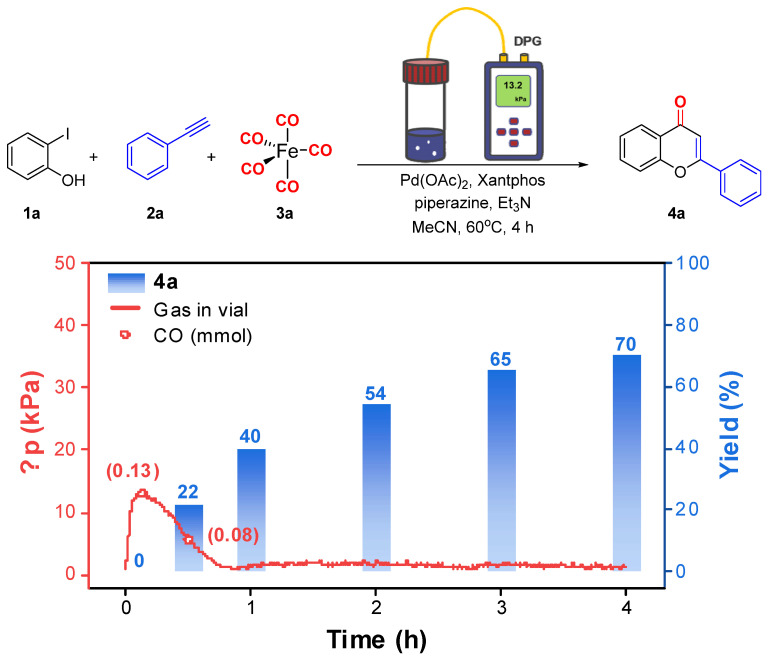
Real-time monitoring of carbon monoxide pressure and quinolone product **4a** in palladium-catalyzed carbonylative cyclization: implications for mechanistic insights.

In the mechanistic investigation, we focused on understanding the dynamics of carbon monoxide (CO) diffusion and transmission in the gas–liquid phase, which is crucial for unraveling the intricacies of the carbonylation mechanism. However, the challenges of handling toxic CO gas and the need for specialized high-pressure nuclear magnetic resonance (HP-NMR) or infrared spectroscopy (IR) equipment present significant hurdles [22]. To address this, we leveraged the unique properties of Fe(CO)_5_ to meticulously examine the kinetics of CO generation and consumption during the palladium-catalyzed carbonylative cyclization process. By integrating a differential pressure gauge (DPG) [23] with NMR technology, we monitored real-time fluctuations in CO pressure alongside the yield of the 4-quinolone product **4a** over a 4 h period, providing valuable insights into the kinetics of carbonylation reactions (Figure 1).

The CO generation rate initially peaked at 7 min, surpassing atmospheric pressure by 13.2 kPa, equivalent to 0.13 mmol of CO molecules. Notably, during the catalytic induction period, the CO released from Fe(CO)_5_ could not be converted to carbonyls, leading to a minor pressure increase due to the escape of CO gas. Subsequently, the gaseous CO content sharply decreased to 0.08 mmol at 0.5 h, while the **4a** product increased to 22%, indicating the presence of approximately 0.435 mmol of CO-containing substances in the liquid phase. The pressure gradually decreased and returned to atmospheric pressure within 1 h, signifying the complete consumption of CO in the gas phase. The yield of **4a** increased to 40% at 1 h, indicating the consumption of 0.2 mmol of CO in the carbonylation cyclization process, leaving 0.425 mmol of CO-containing components in the liquid phase for further conversion.

Differential pressure gauge (DPG) measurements revealed an initial increase followed by a subsequent decrease in the internal pressure, stabilizing after 1 h. NMR analysis demonstrated an increasing yield of the carbonylation product **4a** over time, confirming the conversion of CO into the desired product. The continuous and uniform CO transfer proved to be an effective method to enhance the efficiency of carbonylation cyclization, allowing for a well-balanced equilibrium between the release and consumption rates of CO, resulting in a 70% 4-quinolone yield within 4 h. This approach circumvents the limitations associated with slow gas–liquid mass transfer, offering insights for a new homogenous CO transfer for broader carbonylation applications and mechanistic studies.

**Scheme 3:**
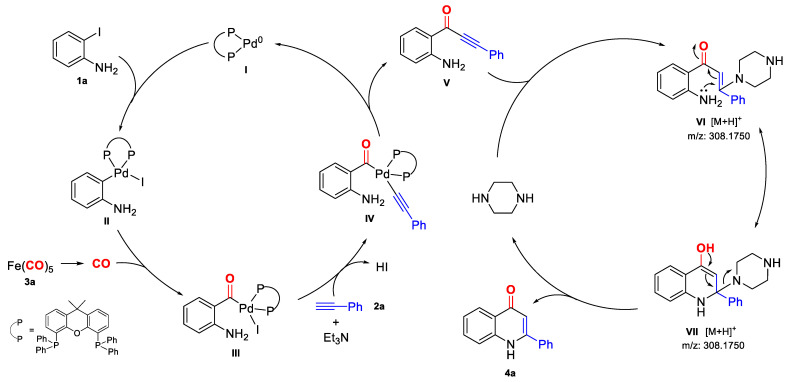
Plausible reaction mechanism for Pd catalyzed synthesis of 4-quinolones. The possible intermediate species in the catalytic cycle serve as I–VII.

Through operando kinetic studies of the Pd-catalyzed carbonylative Sonogashira cyclization, we proposed a homogeneous CO transfer-dominated process between Fe(CO)_5_ and Pd-catalyzed carbonylation reactions, as illustrated in Scheme 3. The product distribution in Table 1 presents compelling evidence of the distinct functions of piperazine and triethylamine in the Pd-catalyzed carbonylative coupling and subsequent cyclization. Notably, the documented enhancement of ynones’ cyclization by piperazine [24] suggests the implementation of a dual-base activation strategy for 4-quinolone synthesis. Triethylamine serves to activate Fe(CO)_5_ and facilitate the in situ release of CO, thereby promoting the carbonylative coupling process. Meanwhile, piperazine contributes to coordinating intramolecular cyclization, highlighting a synergistic role of the dual-base system in the reaction.

The overall reaction can be delineated into two distinct stages: the Pd-catalyzed carbonylative Sonogashira coupling on the left and the subsequent cyclization of the intermediate ynone on the right. Within the left catalytic cycle, mechanistic investigation reveals that the oxidative addition of 2-iodoaniline (**1a**) with the active Xantphos-palladium (0) **I** leads to the formation of palladium complex **II**. The generation of **4a** is concomitant with the detection of only 0.13 mmol of CO gas by DPG, indicating the predominant homogeneous transfer of CO for carbonylation (Figure 1). Intermediate **II** reacts with CO supplied in situ by Fe(CO)_5_/triethylamine, resulting in the formation of acyl palladium complex **III**. The nucleophilic attack of phenylacetylene, facilitated by triethylamine, generates acyl palladium **IV**, followed by the reductive elimination of **IV** to form the carbonylated intermediate **V**.

On the right, piperazine promotes the intramolecular Michael addition of 2-aminoynone **V** to produce **VI**. Subsequently, **VI** further transforms into cyclic enolate **VII**. The characterization of the cyclization intermediate (**VI** or **VII**) with the molecular formula C_19_H_21_N_3_O has been successfully achieved via HR ESI-MS technology, with a calculated *m*/*z* value for [M + H]^+^ of 308.1763 and an observed value of 308.1750. Finally, the piperazine moiety is eliminated from **VI** or **VII** to generate 4-quinolone **4a**. This comprehensive investigation provides detailed insights into the catalytic mechanism, elucidating the specific roles of triethylamine and piperazine involved in the carbonylative Sonogashira cyclization process.

## 3. Experimental

### 3.1. Subsection General Information

All reagents were purchased from the suppliers (Adamas in Basel, Switzerland; Aladdin in Shanghai, China) and used without further purification unless specified otherwise. Flash chromatography was performed using 200–300 mesh silica gel with the indicated solvent system according to standard techniques and analytical thin layer chromatography was carried out using 250 μm commercial silica gel plates. ^1^H NMR and ^13^C NMR were recorded on a Bruker-600 MHz Spectrometer in Billerica, Massachusetts, USA (^1^H: 600 MHz, ^13^C: 151 MHz), using DMSO-d_6_ as the solvent at room temperature. The chemical shifts (δ) were expressed in ppm, and the coupling constants (J) were expressed in Hz. High-resolution mass spectra (HRMS) were recorded on a Bruker MAXIS spectrometer. The change of gas pressure was monitored by differential pressure gauge (Benetech GM522 in Guangdong, China).

### 3.2. General Method for the Synthesis of 4-Quinolones

Pd(OAc)_2_ (5 mol%, 0.025 mmol), 9,9-Dimethyl-4,5-bis(diphenylphosphino)xanthene (10 mol%, 0.05 mmol), 2-iodoaniline (0.5 mmol), and piperazine (1 equiv., 0.5 mmol) were transferred into an oven-dried 31 mL glass vial. After the addition of 4 mL MeCN, terminal alkynes (1.2 equiv., 0.6 mmol), Et_3_N (3 equiv., 1.5 mmol) and iron pentacarbonyl (0.25 equiv., 0.125 mmol) was added. The reaction mixture was stirred at 60 °C for 10 h. After the completion of the reaction, as monitored by TLC, the crude mixture was purified by silica gel column chromatography using dichloromethane (DCM) and methanol as a mixed-solvent system to obtain pure products. All products **4a**–**4v** were identified by comparing their spectral data with those of authentic samples.

2-phenylquinolin-4(1H)-one (**4a**) [25], the general method using aryl iodide **1a** and phenylacetylene **2a**, gave the title compound **4a** in 90% yield; ^1^H NMR (400 MHz, DMSO-*d*_6_) δ 11.74 (s, 1H), 8.12 (d, *J* = 7.8 Hz, 1H), 7.83 (s, 2H), 7.78 (d, *J* = 8.3 Hz, 1H), 7.67 (t, *J* = 7.5 Hz, 1H), 7.57 (s, 3H), 7.34 (t, *J* = 7.3 Hz, 1H), 6.34 (s, 1H); ^13^C NMR (101 MHz, DMSO-*d*_6_) δ 176.98, 149.99, 140.51, 134.20, 131.77, 130.41, 128.96, 127.39, 124.89, 124.71, 123.23, 118.70, 107.34.

2-(m-methoxyphenyl) quinolin-4(1H)-one (**4b**) [26], the general method using aryl iodide **1a** and phenylacetylene **2b**, gave the title compound **4b** in 78% yield; ^1^H NMR (400 MHz, DMSO-*d*_6_) δ 11.74 (s, 1H), 8.13 (d, *J* = 7.9 Hz, 1H), 7.80 (d, *J* = 8.2 Hz, 1H), 7.66 (t, *J* = 7.5 Hz, 1H), 7.48 (t, *J* = 7.7 Hz, 1H), 7.43-7.28 (m, 3H), 7.13 (d, *J* = 7.9 Hz, 1H), 6.39 (s, 1H), 3.86 (s, 3H); ^13^C NMR (101 MHz, DMSO-*d*_6_) δ 177.00, 159.50, 149.80, 140.47, 135.56, 131.74, 130.12, 124.91, 124.70, 123.22, 119.57, 118.72, 116.00, 112.82, 107.40, 55.33.

2-(p-methoxyphenyl) quinolin-4(1H)-one (**4c**) [25], the general method using aryl iodide **1a** and phenylacetylene **2c**, gave the title compound **4c** in 82% yield; ^1^H NMR (400 MHz, DMSO-*d*_6_) δ 11.97 (s, 1H), 8.10 (d, *J* = 7.9 Hz, 1H), 8.02 (d, *J* = 8.3 Hz, 1H), 7.88 (d, *J* = 8.5 Hz, 2H), 7.64 (t, *J* = 7.5 Hz, 1H), 7.31 (t, *J* = 7.4 Hz, 1H), 7.09 (d, *J* = 8.6 Hz, 2H), 6.36 (s, 1H), 3.83 (s, 3H); ^13^C NMR (101 MHz, DMSO-*d*_6_) δ 176.77, 160.98, 149.81, 140.74, 131.43, 128.96, 126.07, 124.66, 124.49, 123.05, 119.01, 114.25, 106.27, 55.39.

2-(m-tolyl) quinolin-4(1H)-one (**4d**) [27], the general method using aryl iodide **1a** and phenylacetylene **2d**, gave the title compound **4d** in 79% yield; ^1^H NMR (400 MHz, DMSO-*d*_6_) δ 11.69 (s, 1H), 8.11 (d, *J* = 7.9 Hz, 1H), 7.78 (d, *J* = 8.3 Hz, 1H), 7.72-7.55 (m, 3H), 7.46 (t, *J* = 7.6 Hz, 1H), 7.42-7.27 (m, 2H), 6.33 (s, 1H), 2.42 (s, 3H); ^13^C NMR (101 MHz, DMSO-*d*_6_) δ 176.90, 150.10, 140.50, 138.33, 134.19, 131.72, 131.00, 128.87, 127.82, 125.83-124.37, 123.19, 118.66, 107.25, 20.96.

2-(p-tolyl) quinoline-4(1H)-one (**4e**) [28], the general method using aryl iodide **1a** and phenylacetylene **2e**, gave the title compound **4e** in 86% yield; ^1^H NMR (400 MHz, DMSO-*d*_6_) δ 12.06 (s, 1H), 8.11 (d, *J* = 7.9 Hz, 1H), 8.01 (d, *J* = 8.3 Hz, 1H), 7.79 (d, *J* = 8.0 Hz, 2H), 7.65 (t, *J* = 7.2 Hz, 1H), 7.33 (dd, *J* = 13.2, 7.7 Hz, 3H), 6.36 (s, 1H), 2.37 (s, 3H); ^13^C NMR (101 MHz, DMSO-*d*_6_) δ 176.92, 150.03, 140.66, 140.22, 131.54, 131.08, 129.42, 127.31, 124.76, 124.53, 123.14, 118.95, 106.78, 20.83.

2-(4-butylphenyl) quinolin-4(1H)-one (**4f**) [14], the general method using aryl iodide **1a** and phenylacetylene **2f**, gave the title compound **4f** in 72% yield; ^1^H NMR (400 MHz, DMSO-*d*_6_) δ 11.63 (s, 1H), 8.10 (d, *J* = 7.9 Hz, 1H), 7.75 (t, *J* = 7.9 Hz, 3H), 7.66 (t, *J* = 7.2 Hz, 1H), 7.40 (d, *J* = 8.0 Hz, 2H), 7.32 (t, *J* = 7.4 Hz, 1H), 6.32 (s, 1H), 2.66 (t, *J* = 7.6 Hz, 2H), 1.66-1.53 (m, 2H), 1.32 (dd, *J* = 14.7, 7.3 Hz, 2H), 0.91 (t, *J* = 7.3 Hz, 3H); ^13^C NMR (101 MHz, DMSO-*d*_6_) δ 176.90, 149.95, 145.09, 140.49, 131.62, 128.88, 127.27, 124.77, 123.13, 118.64, 106.94, 34.51, 32.88, 21.67, 13.71.

2-(2-chlorophenyl) quinolin-4(1H)-one (**4g**) [25], the general method using aryl iodide **1a** and phenylacetylene **2h**, gave the title compound **4g** in 57% yield; ^1^H NMR (400 MHz, DMSO-*d*6) δ 11.98 (s, 1H), 8.14 (d, *J* = 8.0 Hz, 1H), 7.76-7.60 (m, 4H), 7.54 (dt, *J* = 21.2, 7.3 Hz, 2H), 7.35 (t, *J* = 7.3 Hz, 1H), 6.03 (s, 1H); ^13^C NMR (101 MHz, DMSO-*d*6) δ 176.71, 148.10, 140.18, 133.95, 131.93, 131.67, 131.46, 131.12, 129.83, 127.51, 124.86, 124.81, 123.35, 118.45, 109.80.

2-(thiophen-2-yl) quinolin-4(1H)-one (**4h**) [29], the general method using aryl iodide **1a** and phenylacetylene **2i**, gave the title compound **4h** in 73% yield; ^1^H NMR (400 MHz, DMSO-*d*_6_) δ 11.65 (s, 1H), 8.09 (d, *J* = 7.7 Hz, 1H), 7.82 (dd, *J* = 38.9, 17.2 Hz, 3H), 7.67 (t, *J* = 7.3 Hz, 1H), 7.31 (d, *J* = 17.1 Hz, 2H), 6.34 (s, 1H); ^13^C NMR (101 MHz, DMSO-*d*_6_) δ 176.70, 143.53, 140.30, 136.09, 131.97, 129.64, 128.55, 128.20, 124.92, 124.68, 123.29, 118.50, 106.13.

2-(pyridin-3-yl)quinolin-4(1H)-one (**4i**) [29], the general method using aryl iodide **1a** and phenylacetylene **2j**, gave the title compound **4i** in 79% yield; ^1^H NMR (600 MHz, DMSO-*d*_6_) δ 11.91 (s, 1H), 9.05 (s, 1H), 8.76 (d, *J* = 4.7 Hz, 1H), 8.27 (d, *J* = 7.9 Hz, 1H), 8.12 (d, *J* = 8.1 Hz, 1H), 7.76 (dd, *J* = 8.6, 4.2 Hz, 1H), 7.70 (t, *J* = 7.6 Hz, 1H), 7.63 (dd, *J* = 8.0, 4.9 Hz, 1H), 7.37 (t, *J* = 7.5 Hz, 1H), 6.46 (s, 1H). ^13^C NMR (151 MHz, DMSO-*d*_6_) δ 177.40, 151.64, 148.60, 147.88, 141.01, 135.71, 132.46, 130.56, 125.38, 125.25, 124.29, 123.93, 119.20, 108.43.

2-hexylquinolin-4(1H)-one (**4j**) [5], the general method using aryl iodide **1a** and phenylacetylene **2k**, gave the title compound **4j** in 43% yield; ^1^H NMR (400 MHz, DMSO-*d*_6_) δ 11.68 (s, 1H), 8.05 (d, *J* = 8.1 Hz, 1H), 7.62 (t, *J* = 7.6 Hz, 1H), 7.56 (d, *J* = 8.3 Hz, 1H), 7.30 (q, *J* = 7.4, 6.8 Hz, 1H), 5.98 (s, 1H), 2.60 (t, *J* = 7.7 Hz, 2H), 1.66 (p, *J* = 7.4 Hz, 2H), 1.29 (q, *J* = 6.6, 5.9 Hz, 6H), 0.90–0.79 (m, 3H). ^13^C NMR (101 MHz, DMSO-*d*_6_) δ 176.47, 153.95, 140.11, 131.53, 124.65, 124.33, 122.91, 117.95, 107.45, 33.24, 30.89, 28.30, 28.13, 21.94, 13.85.

2-heptylquinolin-4(1H)-one (**4k**) [30], the general method using aryl iodide **1a** and phenylacetylene **2l**, gave the title compound **4k** in 47% yield; ^1^H NMR (600 MHz, DMSO-*d*_6_) δ 11.62 (s, 1H), 8.05 (d, *J* = 8.0 Hz, 1H), 7.59 (d, *J* = 6.8 Hz, 1H), 7.55 (d, *J* = 8.2 Hz, 1H), 7.26 (t, *J* = 7.4 Hz, 1H), 5.94 (s, 1H), 2.56 (t, *J* = 7.7 Hz, 2H), 1.64 (p, *J* = 7.3 Hz, 2H), 1.31–1.14 (m, 8H), 0.81 (t, *J* = 6.8 Hz, 3H). ^13^C NMR (151 MHz, DMSO-*d*_6_) δ 177.32, 154.13, 140.65, 131.88, 125.20, 125.05, 123.19, 118.38, 108.05, 33.73, 31.63, 28.96, 28.87, 22.52, 14.35.

6-chloro-2-phenylquinolin-4(1H)-one (**4l**) [14], the general method using aryl iodide **1b** and phenylacetylene **2a**, gave the title compound **4l** in 51% yield; ^1^H NMR (400 MHz, DMSO-*d*_6_) δ 11.85 (s, 1H), 8.03 (s, 1H), 7.83 (s, 3H), 7.72 (s, 1H), 7.59 (s, 3H), 6.38 (s, 1H); ^13^C NMR (101 MHz, DMSO-*d*_6_) δ 175.69, 150.37, 139.10, 133.90, 131.91, 130.60, 129.01, 127.91, 127.42, 125.88, 123.66, 121.17, 107.49.

6-chloro-2-(4-methoxyphenyl) quinolin-4(1H)-one (**4m**) [31], the general method using aryl iodide **1b** and phenylacetylene **2c**, gave the title compound **4m** in 60% yield; ^1^H NMR (600 MHz, DMSO-*d*_6_) δ 11.76 (s, 1H), 8.01 (s, 1H), 7.81 (d, *J* = 8.3 Hz, 3H), 7.70 (d, *J* = 8.7 Hz, 1H), 7.14 (d, *J* = 8.5 Hz, 2H), 6.35 (s, 1H), 3.85 (s, 3H); ^13^C NMR (151 MHz, DMSO-*d*_6_) δ 175.57, 161.18, 150.03, 139.08, 131.74, 128.89, 127.71, 125.85, 125.80, 123.60, 121.06, 114.41, 106.62, 55.44.

6-chloro-2-(p-tolyl) quinolin-4(1H)-one (**4n**) [32], the general method using aryl iodide **1b** and phenylacetylene **2e**, gave the title compound **4n** in 62% yield; ^1^H NMR (600 MHz, DMSO-*d*_6_) δ 11.78 (s, 1H), 8.02 (s, 1H), 7.80 (d, *J* = 8.3 Hz, 1H), 7.78-7.66 (m, 3H), 7.40 (d, *J* = 6.2 Hz, 2H), 6.37 (s, 1H), 2.41 (s, 3H); ^13^C NMR (151 MHz, DMSO-*d*_6_) δ 175.66, 150.29, 140.57, 139.10, 131.84, 130.96, 129.57, 127.82, 127.24, 125.88, 123.65, 121.12, 107.08, 20.87.

7-methyl-2-phenylquinolin-4(1H)-one (**4o**) [10], the general method using aryl iodide **1c** and phenylacetylene **2a**, gave the title compound **4o** in 92% yield; ^1^H NMR (400 MHz, DMSO-*d*_6_) δ 11.57 (s, 1H), 8.00 (d, *J* = 8.0 Hz, 1H), 7.81 (d, *J* = 3.5 Hz, 2H), 7.56 (d, *J* = 12.2 Hz, 4H), 7.16 (d, *J* = 7.9 Hz, 1H), 6.28 (s, 1H), 2.44 (s, 4H); ^13^C NMR (101 MHz, DMSO-*d*_6_) δ 176.80, 149.74, 141.82, 140.68, 134.27, 130.33, 128.95, 127.34, 124.90, 124.70, 122.90, 117.96, 107.22, 21.37.

7-methyl-2-(m-methoxyphenyl) quinolin-4(1H)-one (**4p**), the general method using aryl iodide **1c** and phenylacetylene **2b**, gave the title compound **4p** in 82% yield; ^1^H NMR (400 MHz, DMSO-*d*_6_) δ 11.54 (s, 1H), 7.98 (d, *J* = 8.2 Hz, 1H), 7.50 (dd, *J* = 17.6, 9.7 Hz, 2H), 7.41-7.32 (m, 2H), 7.15 (t, *J* = 8.2 Hz, 2H), 6.31 (s, 1H), 3.87 (s, 3H), 2.44 (s, 3H); ^13^C NMR (101 MHz, DMSO-*d*_6_) δ 176.80, 159.51, 149.53, 141.82, 140.62, 135.64, 130.14, 124.92, 124.69, 122.92, 119.53, 117.97, 116.02, 112.72, 107.27, 55.36, 21.37. HRMS (ESI-TOF) *m*/*z*: [M + H]^+^ Calcd. For C_17_H_16_NO_2_^+^: 266.1181, Found: 266.1171.

7-methyl-2-(p-methoxyphenyl) quinolin-4(1H)-one (**4q**), the general method using aryl iodide **1c** and phenylacetylene **2c**, gave the title compound **4q** in 85% yield; ^1^H NMR (400 MHz, DMSO-*d*_6_) δ 11.42 (s, 1H), 7.97 (d, *J* = 8.0 Hz, 1H), 7.78 (d, *J* = 7.9 Hz, 2H), 7.53 (s, 1H), 7.13 (d, *J* = 7.0 Hz, 3H), 6.25 (s, 1H), 3.85 (s, 4H), 2.43 (s, 3H); ^13^C NMR (101 MHz, DMSO-*d*_6_) δ 176.70, 160.98, 149.40, 141.64, 140.65, 128.75, 126.31, 124.72, 124.67, 122.81, 117.86, 114.36, 106.38, 55.42, 21.37. HRMS (ESI-TOF) *m*/*z*: [M + H]^+^ Calcd. For C_17_H_16_NO_2_^+^: 266.1181, Found: 266.1168.

7-methyl-2-(m-tolyl) quinolin-4(1H)-one (**4r**), the general method using aryl iodide **1c** and phenylacetylene **2d**, gave the title compound **4r** in 88% yield; ^1^H NMR (400 MHz, DMSO-*d*_6_) δ 11.54 (s, 1H), 7.98 (d, *J* = 8.2 Hz, 1H), 7.65-7.57 (m, 2H), 7.53 (s, 1H), 7.46 (t, *J* = 7.6 Hz, 1H), 7.38 (d, *J* = 7.3 Hz, 1H), 7.15 (d, *J* = 8.1 Hz, 1H), 6.26 (s, 1H), 2.43 (d, *J* = 5.3 Hz, 6H); ^13^C NMR (101 MHz, DMSO-*d*_6_) δ 176.77, 149.84, 141.78, 140.66, 138.32, 134.24, 130.94, 128.87, 127.79, 124.86, 124.69, 124.46, 122.90, 117.94, 107.14, 21.38, 20.97. HRMS (ESI-TOF) *m*/*z*: [M + H]^+^ Calcd. For C_17_H_16_NO^+^: 250.1232, Found: 250.1222.

7-methyl-2-(p-tolyl) quinolin-4(1H)-one (**4s**), the general method using aryl iodide **1c** and phenylacetylene **2e**, gave the title compound **4s** in 86% yield; ^1^H NMR (400 MHz, DMSO-*d*_6_) δ 11.48 (s, 1H), 7.97 (d, *J* = 8.2 Hz, 1H), 7.72 (d, *J* = 7.8 Hz, 2H), 7.53 (s, 1H), 7.39 (d, *J* = 7.8 Hz, 2H), 7.15 (d, *J* = 8.2 Hz, 1H), 6.26 (s, 1H), 2.44 (s, 3H), 2.40 (s, 3H); ^13^C NMR (101 MHz, DMSO-*d*_6_) δ 176.76, 140.66, 140.22, 131.34, 129.50, 127.15, 124.81, 124.68, 117.91, 106.80. HRMS (ESI-TOF) *m*/*z*: [M + H]^+^ Calcd. For C_17_H_16_NO^+^: 250.1232, Found: 250.1221.

7-methyl-2-(4-butylphenyl) quinolin-4(1H)-one (**4t**), the general method using aryl iodide **1c** and phenylacetylene **2f**, gave the title compound **4t** in 76% yield; ^1^H NMR (400 MHz, DMSO-*d*_6_) δ 11.48 (s, 1H), 7.98 (d, *J* = 8.1 Hz, 1H), 7.72 (d, *J* = 7.9 Hz, 2H), 7.53 (s, 1H), 7.39 (d, *J* = 7.9 Hz, 2H), 7.15 (d, *J* = 8.1 Hz, 1H), 6.26 (s, 1H), 2.67 (t, *J* = 7.6 Hz, 2H), 2.43 (s, 3H), 1.66-1.54 (m, 2H), 1.34 (dt, *J* = 14.6, 7.2 Hz, 2H), 0.91 (t, *J* = 7.3 Hz, 3H); ^13^C NMR (151 MHz, DMSO-*d*_6_) δ 176.79, 149.75, 145.03, 141.77, 140.65, 131.61, 128.88, 127.23, 124.84, 124.69, 122.86, 117.91, 106.83, 34.50, 32.88, 21.67, 21.37, 13.71. HRMS (ESI-TOF) *m*/*z*: [M + H]^+^ Calcd. For C_20_H_22_NO^+^: 292.1701, Found: 292.1690.

7-methyl-2-(4-(4-oxo-1,4-dihydroquinolin-2-yl) phenyl) acetonitrile (**4u**), the general method using aryl iodide **1c** and phenylacetylene **2g**, gave the title compound **4u** in 58% yield; ^1^H NMR (600 MHz, DMSO-*d*_6_) δ 11.58 (s, 1H), 7.99 (d, *J* = 8.2 Hz, 1H), 7.85 (d, *J* = 7.7 Hz, 2H), 7.58-7.50 (m, 3H), 7.16 (d, *J* = 7.9 Hz, 1H), 6.30 (s, 1H), 4.16 (s, 2H), 2.44 (s, 3H); ^13^C NMR (151 MHz, DMSO-*d*_6_) δ 176.78, 149.11, 141.90, 140.66, 133.62, 128.66, 127.93, 124.96, 124.71, 122.91, 118.94, 117.94, 107.26, 22.23, 21.38. HRMS (ESI-TOF) *m*/*z*: [M + H]^+^ Calcd. For C_18_H_15_N_2_O^+^: 275.1184, Found: 275.1177.

7-methyl-2-(thiophen-2-yl) quinolin-4(1H)-one (**4v**), the general method using aryl iodide **1c** and phenylacetylene **2i**, gave the title compound **4v** in 78% yield; ^1^H NMR (400 MHz, DMSO-*d*_6_) δ 11.50 (s, 1H), 7.96 (d, *J* = 8.0 Hz, 1H), 7.86 (d, *J* = 19.7 Hz, 2H), 7.53 (s, 1H), 7.28 (s, 1H), 7.15 (d, *J* = 7.2 Hz, 1H), 6.27 (s, 1H), 2.44 (s, 3H); ^13^C NMR (101 MHz, DMSO-*d*_6_) δ 176.56, 143.28, 142.09, 140.44, 136.17, 129.52, 128.55, 128.06, 124.95, 124.66, 122.93, 117.80, 106.00, 99.49, 21.35. HRMS (ESI-TOF) *m*/*z*: [M + H]^+^ Calcd. For C_14_H_12_NOS^+^: 242.0640, Found: 242.0630.

## 4. Conclusions

In summary, we have established a robust and efficient method for synthesizing 4-quinolone compounds under mild reaction conditions. The successful coordination of three processes, CO release from Fe(CO)_5_, Pd-catalyzed carbonylation in Sonogashira coupling, and intramolecular cyclization, has been achieved through the regulation of a dual-base system comprising piperazine and triethylamine. This precise control facilitated the carbonylative cyclization reaction of 2-iodoaniline and terminal alkynes, leading to the synthesis of 22 different 4-quinolone drugs. Under sub-mol Fe(CO)_5_ as the carbonyl source, the efficient conversion of in situ CO to the carbonyl-containing products was achieved. Additionally, the monitoring experiments of pressure verified the homogeneous and controllable CO transfer, providing a valuable reference for broader carbonylation applications and mechanistic studies.

## Figures and Tables

**Table 1:** Screening the reaction conditions.

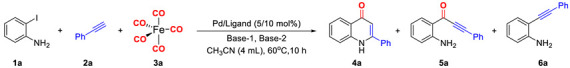							
Entry *^a^*	Pd	Ligand	Base-1 (3 Equiv.)	Base-2 (1 Equiv.)	Yield (%) *^b^*		
					4a	5a	6a
1	Pd(OAc)_2_	-	Piperazine	-	57	-	32
2	Pd(OAc)_2_	-	K_2_CO_3_	-	41	-	15
3	Pd(OAc)_2_	-	K_3_PO_4_	-	29	-	20
4	Pd(OAc)_2_	-	Et_3_N	-	53	3	4
5	Pd(OAc)_2_	-	TMEDA	-	25	12	3
6	Pd(OAc)_2_	-	Piperazine	Et_3_N	70	-	21
7	Pd(OAc)_2_	-	Et_3_N	Piperazine	85	-	10
8	Pd_2_(dba)_3_	-	Et_3_N	Piperazine	63	8	21
9	Pd(OAc)_2_	PPh_3_	Et_3_N	Piperazine	66	8	26
10	Pd(OAc)_2_	dppp	Et_3_N	Piperazine	88	7	5
11	Pd(OAc)_2_	Xantphos	Et_3_N	Piperazine	97	-	3
12 *^c^*	Pd(OAc)_2_	Xantphos	Et_3_N	Piperazine	90	-	10
13 *^d^*	Pd(OAc)_2_	Xantphos	Et_3_N	Piperazine	78	-	22

*^a^* Reaction conditions: 2-iodoaniline (0.5 mmol), Phenylacetylene (1.2 equiv.), [Pd] (5 mol%), Ligand (10 mol%), Base-1 (3.0 equiv.), Base-2 (1.0 equiv.), Fe(CO)_5_ (0.5 equiv.), and CH_3_CN (4 mL) was stirred at 60 °C for 10 h. *^b^* The yields were determined by ^1^H NMR using 3,4,5-trichloropyridine as the internal standard. *^c^* Fe(CO)_5_ (0.25 equiv.). *^d^* Fe(CO)_5_ (0.2 equiv.).

**Table 2:** Three-component Pd-catalyzed carbonylative cyclization for the synthesis of **4a**.

Entry	CO/CORM	Conditions	4a-Yield (%)
1 [5]	CO (20 bar)	PdCl_2_(PPh_3_)_2_, Et_2_NH, 120 °C, 6 h	90
2 [7]	CO (5 bar)	PdCl_2_(dppp), toluene, Et_3_N, 80 °C, 6 h	62
		b. Et_2_NH, 1 h	
3 [12]	Mo(CO)_6_ (2 equiv.)	Pd_2_(dba)_3_, dppf, Et_2_NH, MW 120 °C, 20 min	85
4 [12]	Mo(CO)_6_ (1.5 equiv.)	Pd(OAc)_2_, [HP(*t*Bu)_3_]BF_4_, MeCN, Et_3_N, r.t., 16 h	84
		Et_2_NH, 5 h	
5	Fe(CO)_5_ (0.25 equiv.)	Pd(OAc)_2_, Xantphos, piperazine, Et_3_N, CH_3_CN, 60 °C 10 h	91

## Data Availability

Data are contained within the article and Supplementary Materials.

## Supplementary Materials

The following supporting information can be downloaded at: https://www.mdpi.com/article/10.3390/molecules29040850/s1, This file contains details about the spectrum of the products.
