# Supplementary material for: Enhanced Selectivity in 4-Quinolone Formation: A Dual-Base System for Palladium-Catalyzed Carbonylative Cyclization with Fe(CO)5
Source: Molecules. 2024 Feb 14;29(4):850. doi: 10.3390/molecules29040850 (PMC10892599; doi:10.3390/molecules29040850)

Supplementary Materials

Enhanced Selectivity in 4-Quinolone Formation: A Dual-Base System for Palladium-Catalyzed Carbonylative Cyclization with $Fe(CO)_5$

Meng Guo, Dou Wu, Hongyu Yang, Xiao Zhang, Dong-Xu Xue*, and Weiqiang Zhang*

E-mail: xuedx@snnu.edu.cn and zwq@snnu.edu.cn

Contents

1. Real-time monitoring of carbon monoxide pressure 1
2. ^{1}H NMR and ^{13}C NMR Spectra 2
3. HRMS Spectra 24

1. Real-time monitoring of carbon monoxide pressure

The Sonogashira carbonylative annulation of 2-iodoaniline, phenylacetylene, and $Fe(CO)_5$ was used as the model reaction. Under the identified reaction conditions (**Table 1**, entry 12), we observed pressure changes in the reaction vial (**Figure 1**). All reagents were added to a 31 mL vial at room temperature, then a stirrer was added and the vial was sealed with a rubber stopper. The differential pressure gauge was connected and the needle hole was sealed with hot melt adhesive. After preparation, the reaction vial was immediately placed on a stirring table heated to 60℃ and the speed was adjusted to 400 rpm. Pressure changes were recorded continuously for 4 hours, and the results are shown in **Figure 1**.

2. ^{1}H NMR and ^{13}C NMR Spectra

2-phenylquinolin-4(1H)-one (4a) ^{1}H NMR

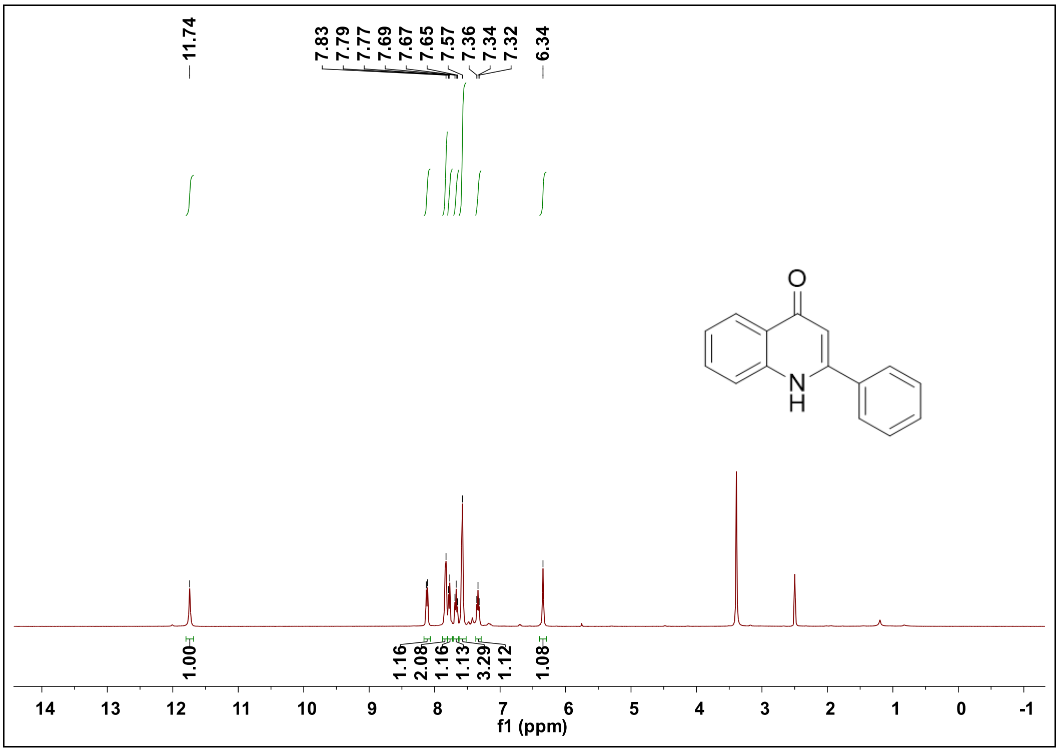

2-phenylquinolin-4(1H)-one (4a) ^{13}C NMR

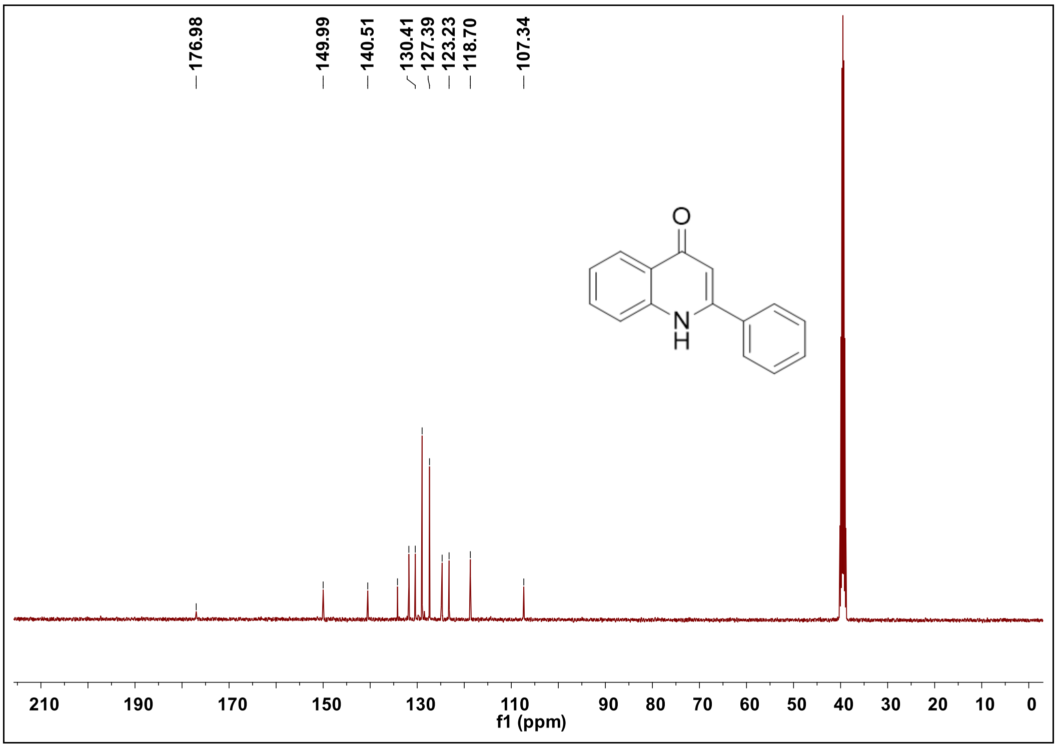

2-(m-methoxyphenyl) quinolin-4(1H)-one (4b) ^1H NMR

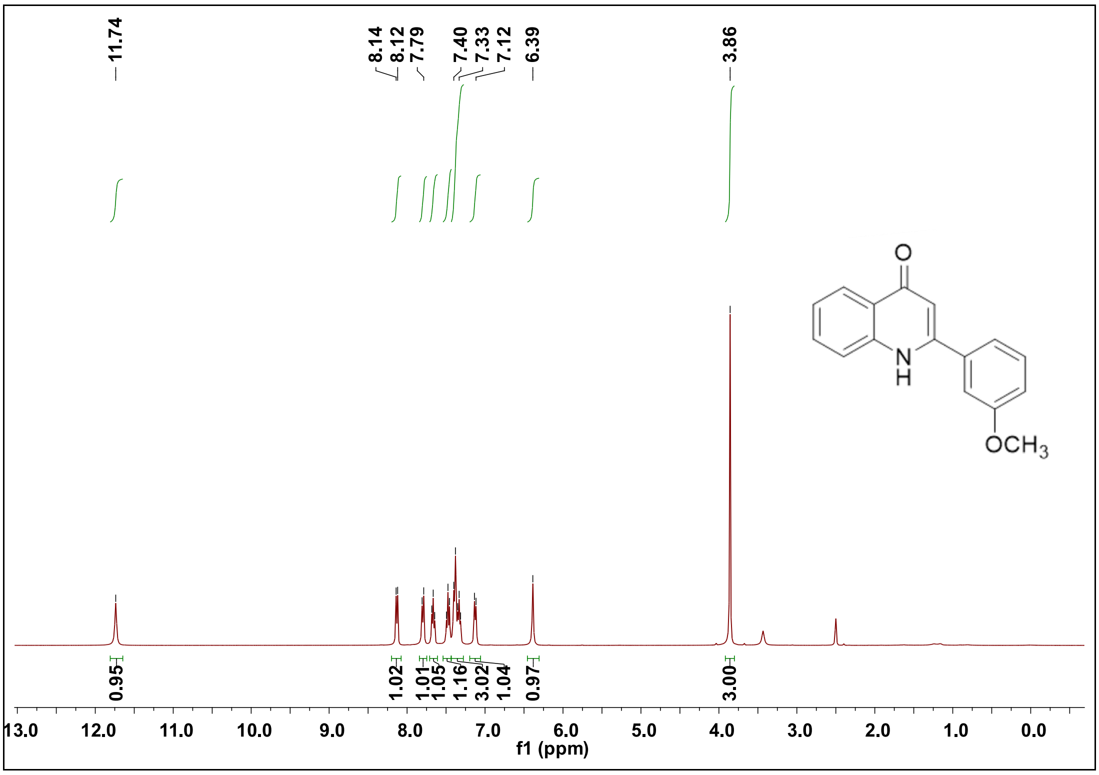

2-(m-methoxyphenyl) quinolin-4(1H)-one (4b) ^{13}C NMR

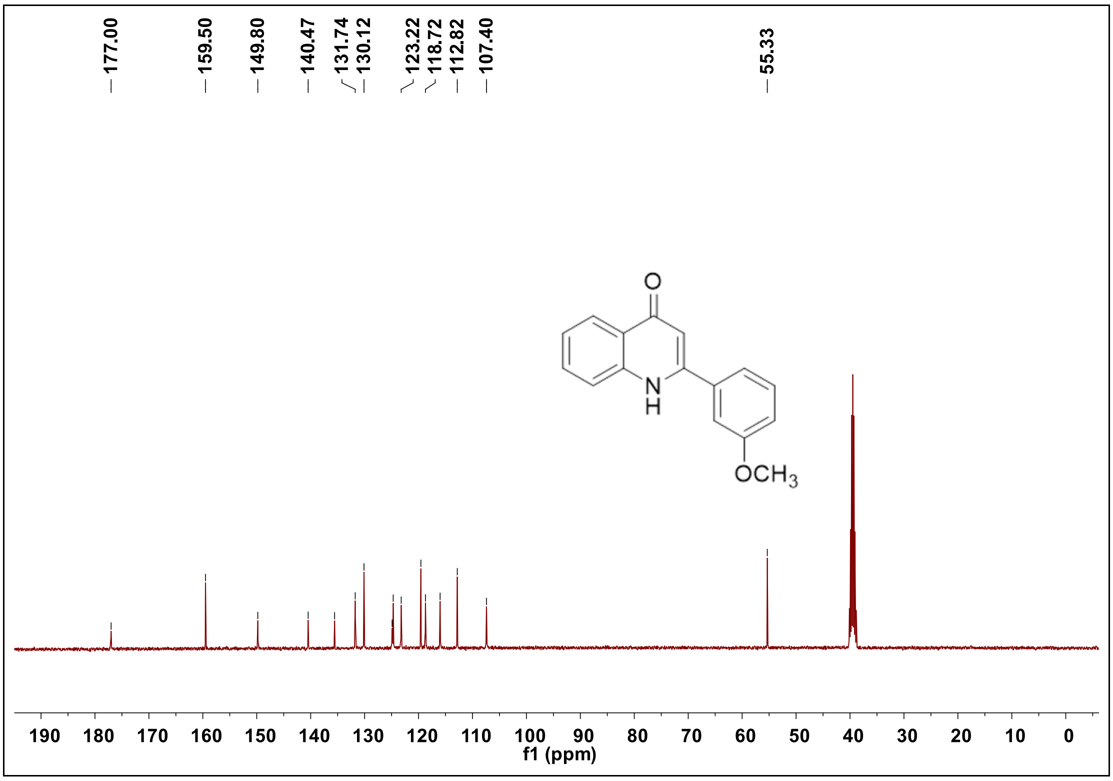

2-(p-methoxyphenyl) quinolin-4(1H)-one (4c) ¹H NMR

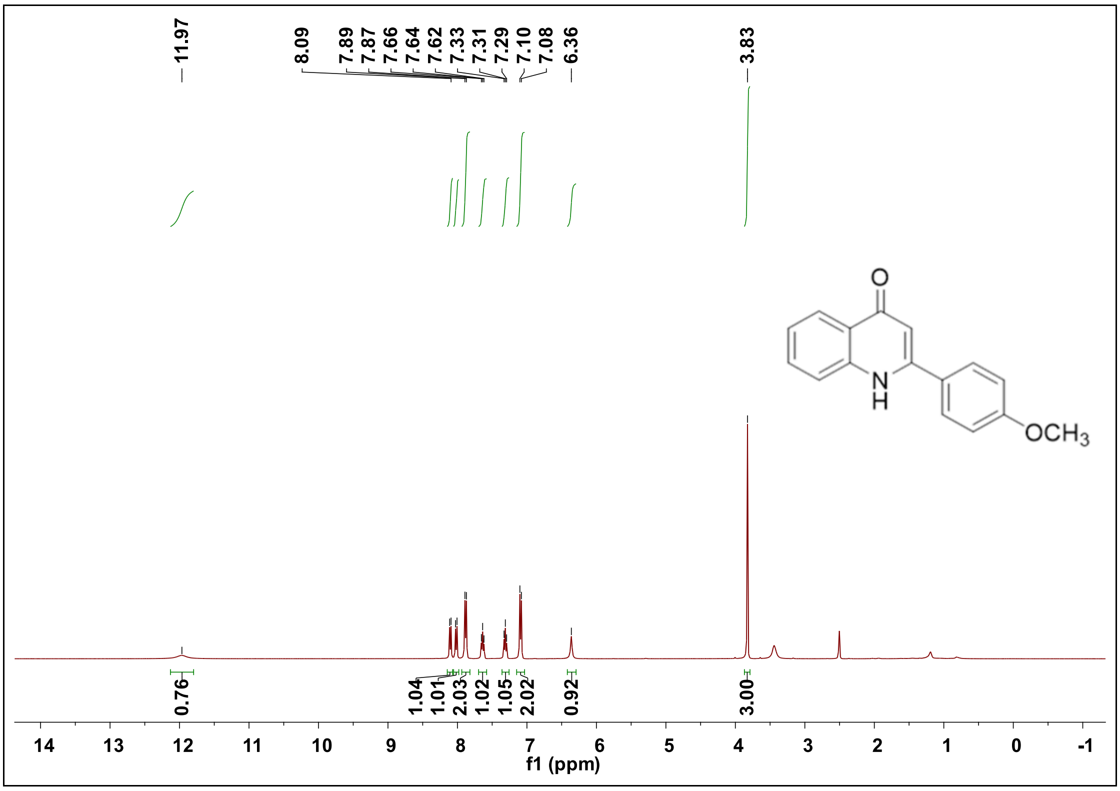

2-(p-methoxyphenyl) quinolin-4(1H)-one (4c) ¹³C NMR

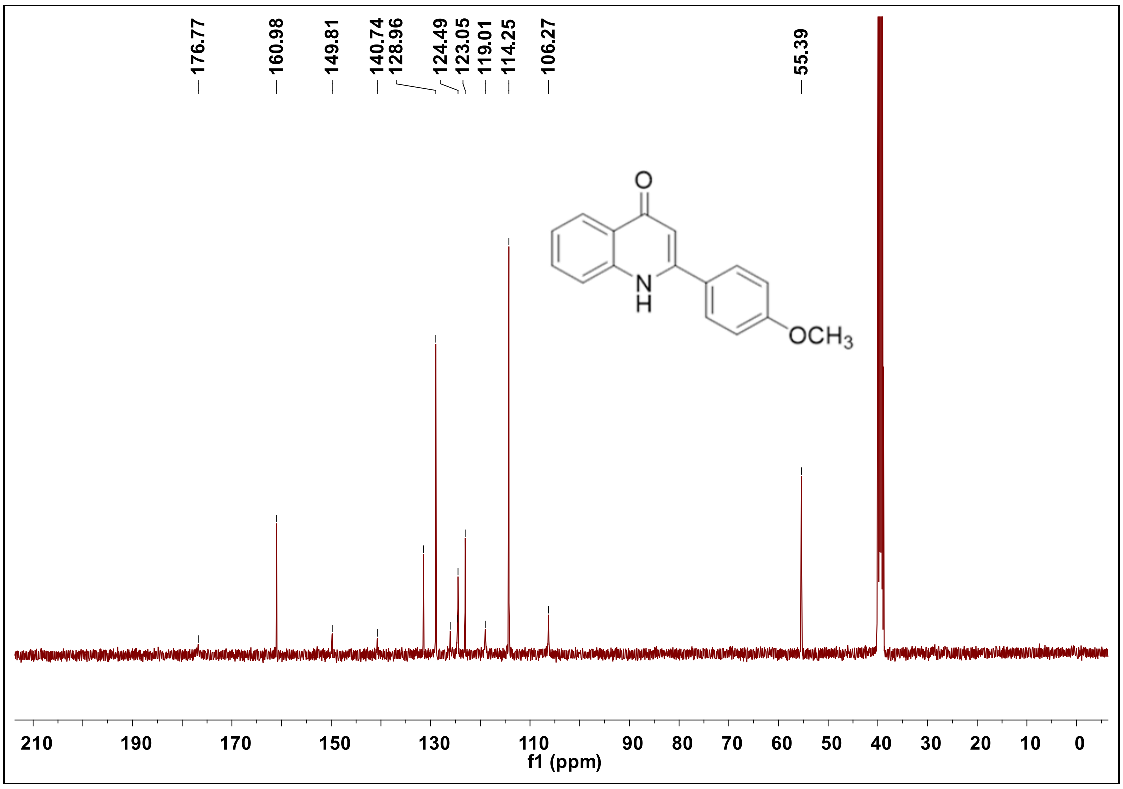

2-(m-tolyl) quinolin-4(1H)-one (4d) ^{1}H NMR

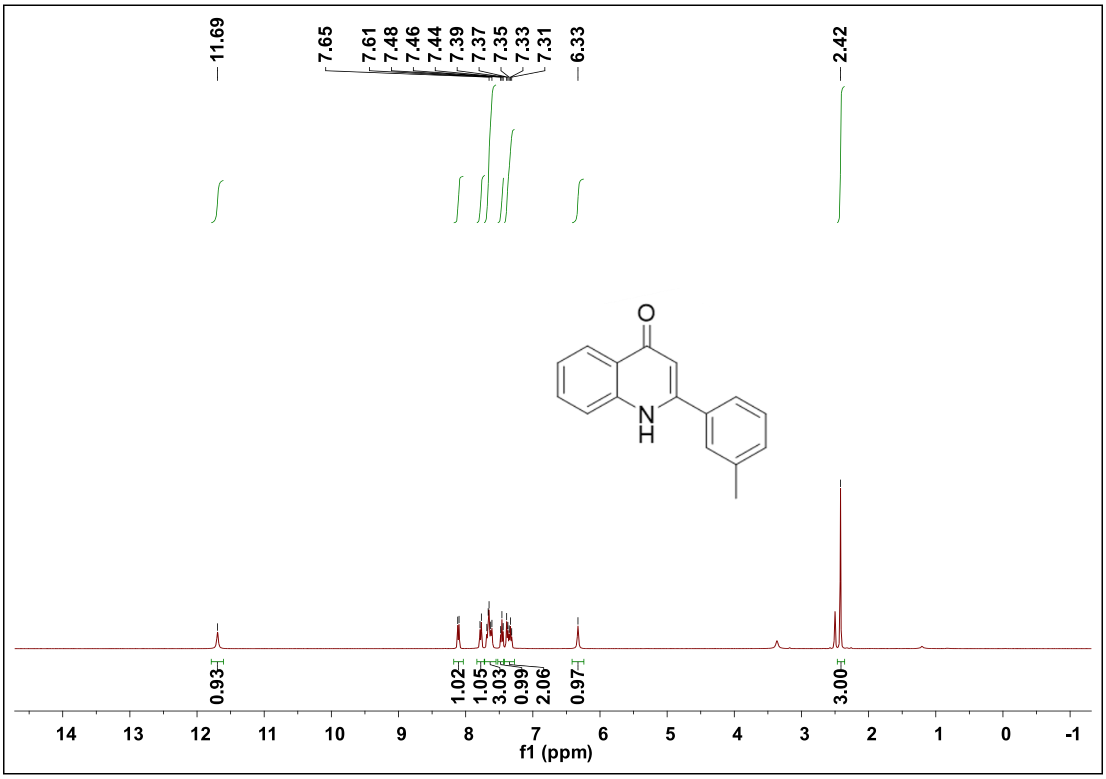

2-(m-tolyl) quinolin-4(1H)-one (4d) ^{13}C NMR

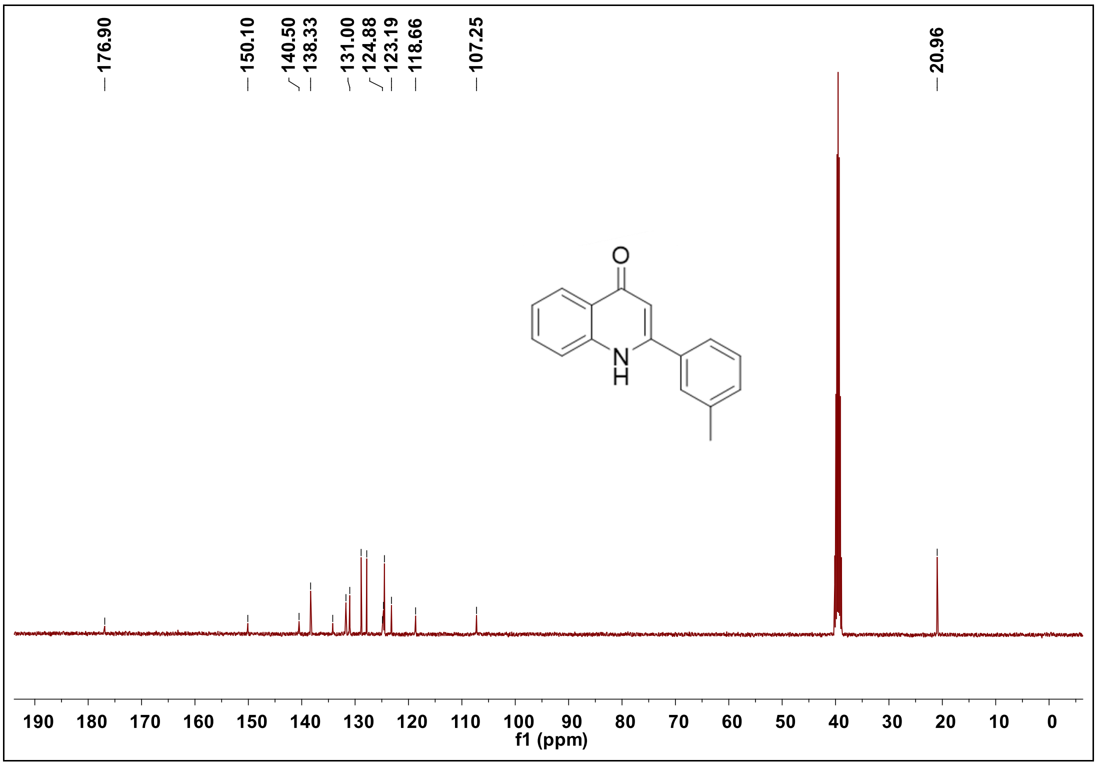

2-(p-tolyl) quinolin-4(1H)-one (4e) ^{1}H NMR

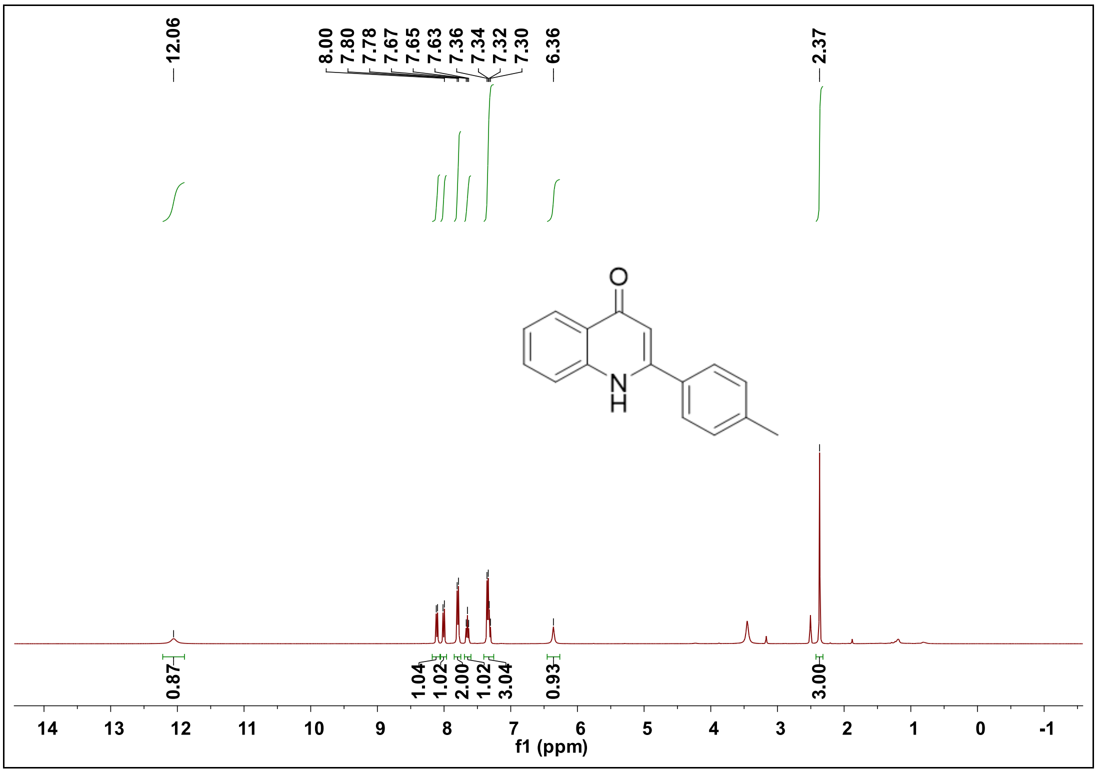

2-(p-tolyl) quinolin-4(1H)-one (4e) ^{13}C NMR

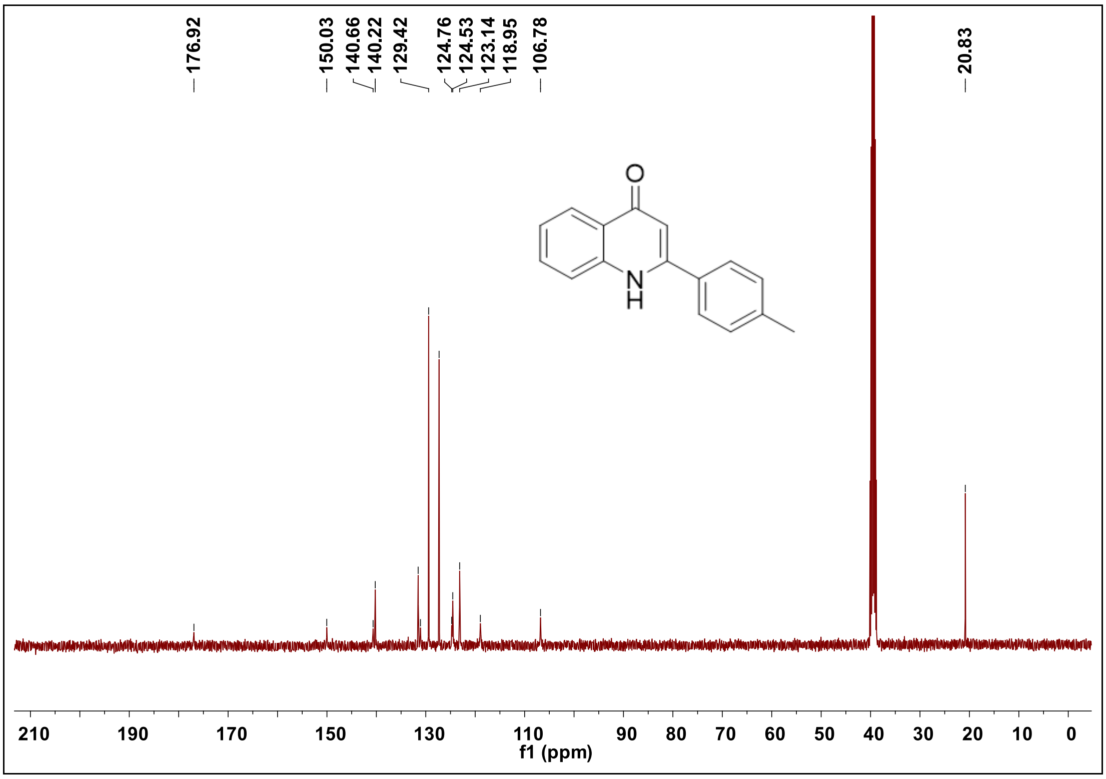

2-(4-butylphenyl) quinolin-4(1H)-one (4f) ^{1}H NMR

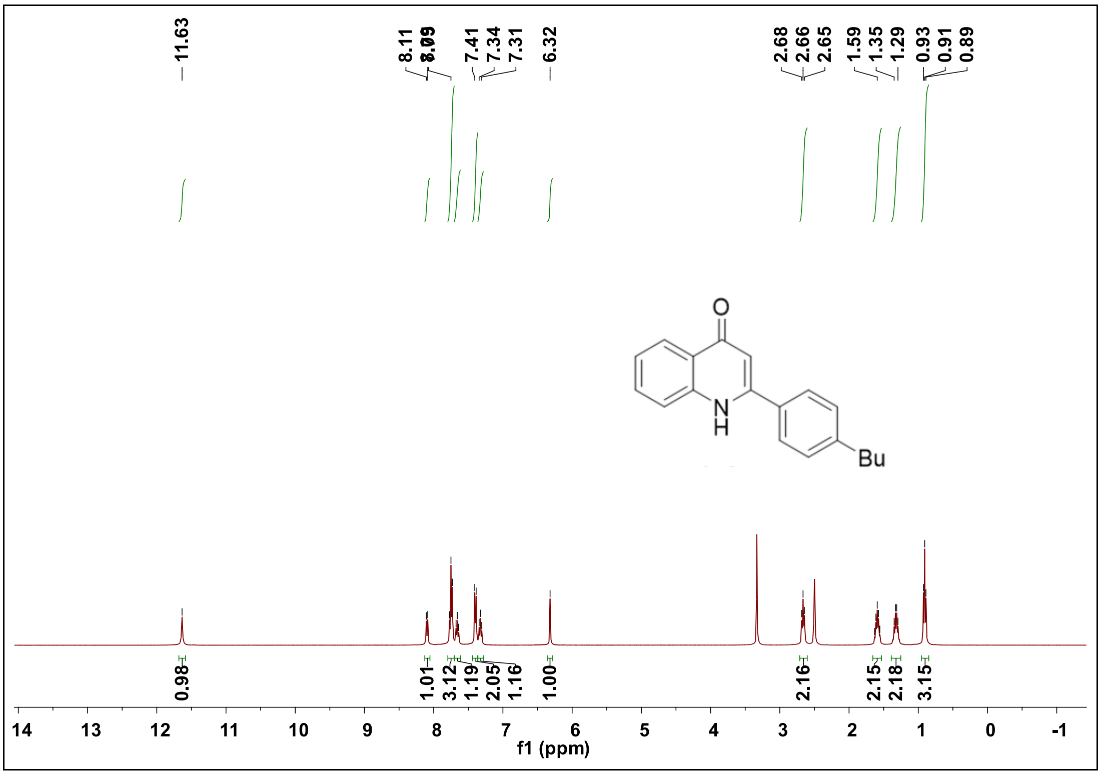

2-(4-butylphenyl) quinolin-4(1H)-one (4f) ^{13}C NMR

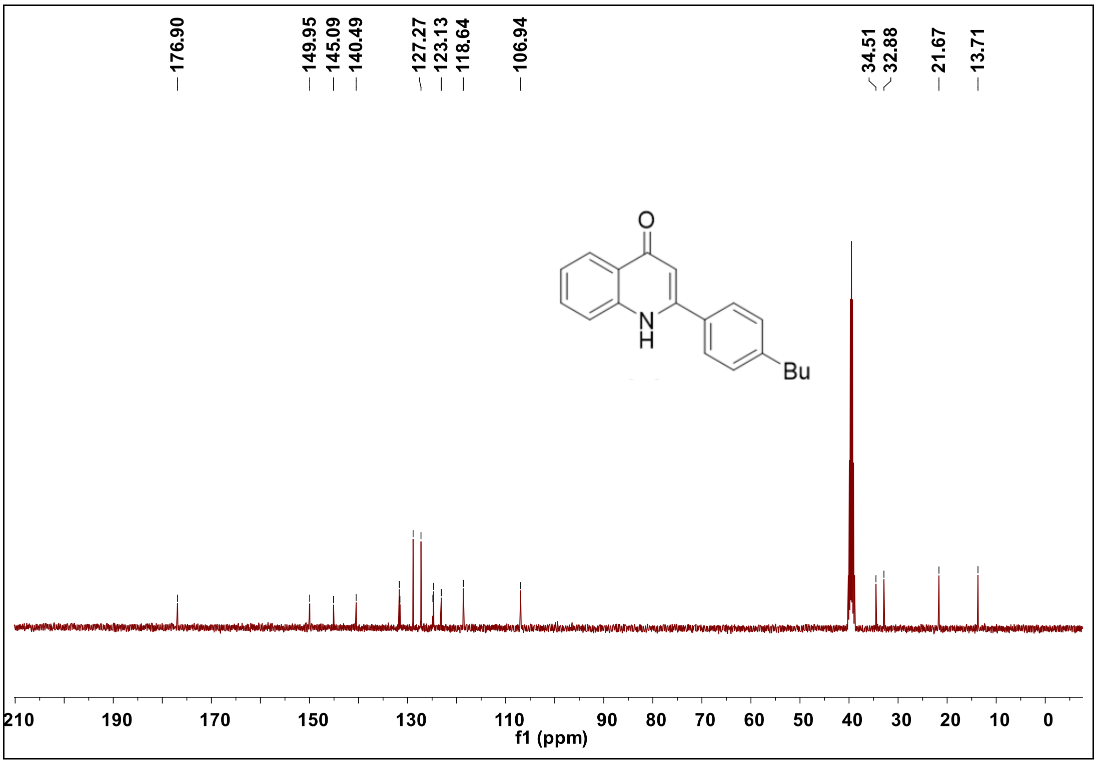

2-(2-chlorophenyl) quinolin-4(1H)-one (4g) ^{1}H NMR

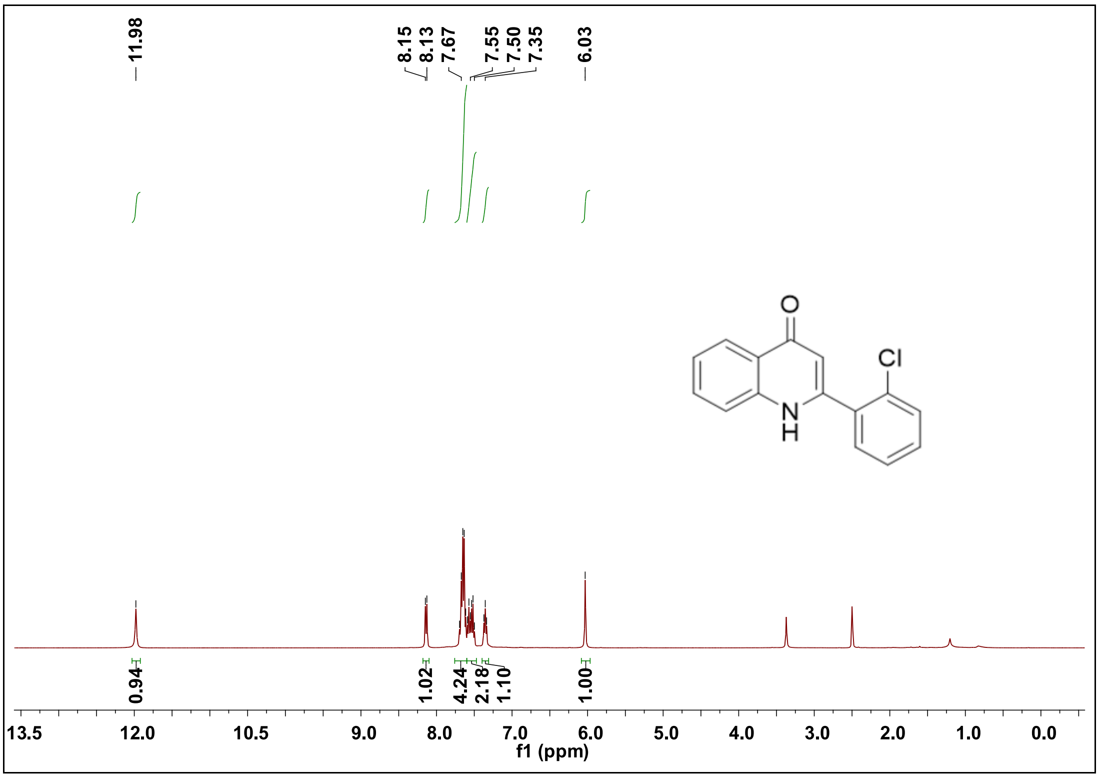

2-(2-chlorophenyl) quinolin-4(1H)-one (4g) ^{13}C NMR

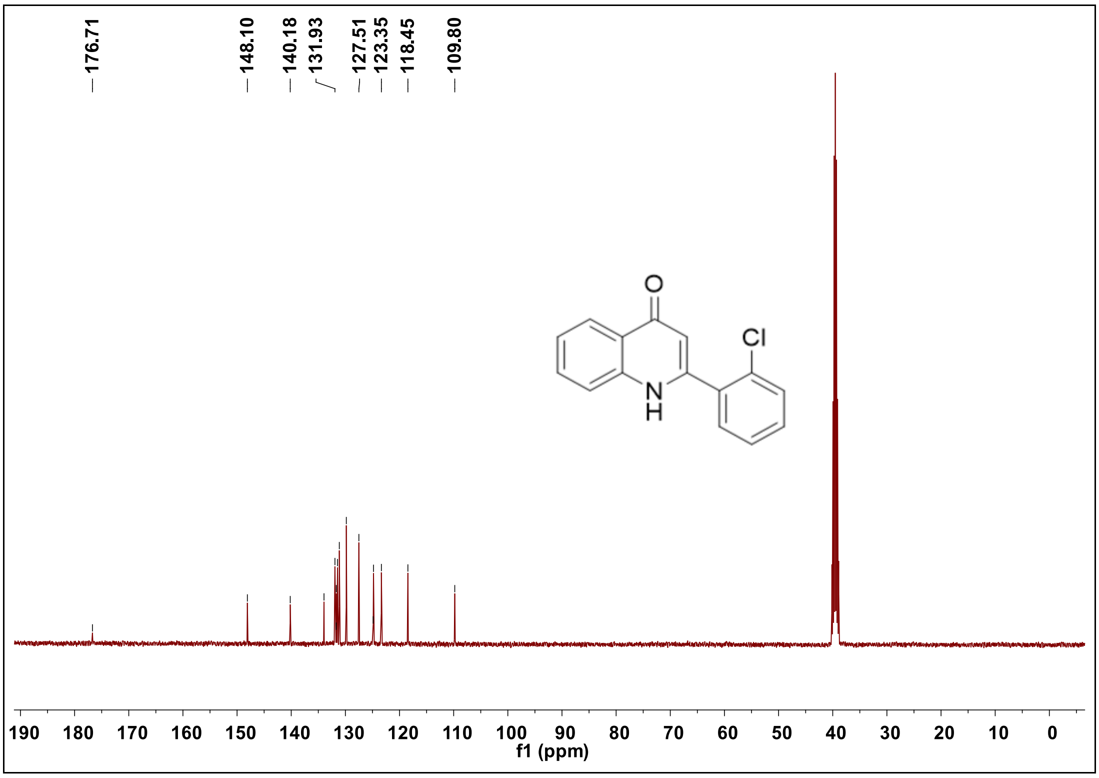

2-(thiophen-2-yl) quinolin-4(1H)-one (4h) ^{1}H NMR

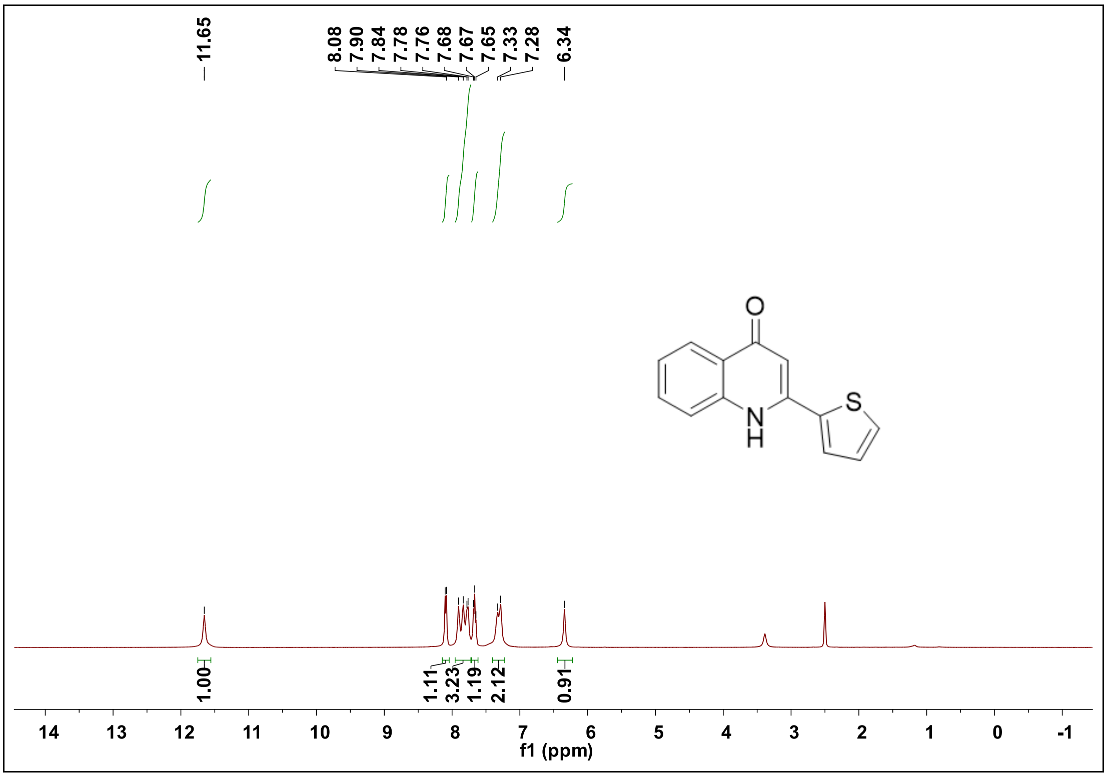

2-(thiophen-2-yl) quinolin-4(1H)-one (4h) ^{13}C NMR

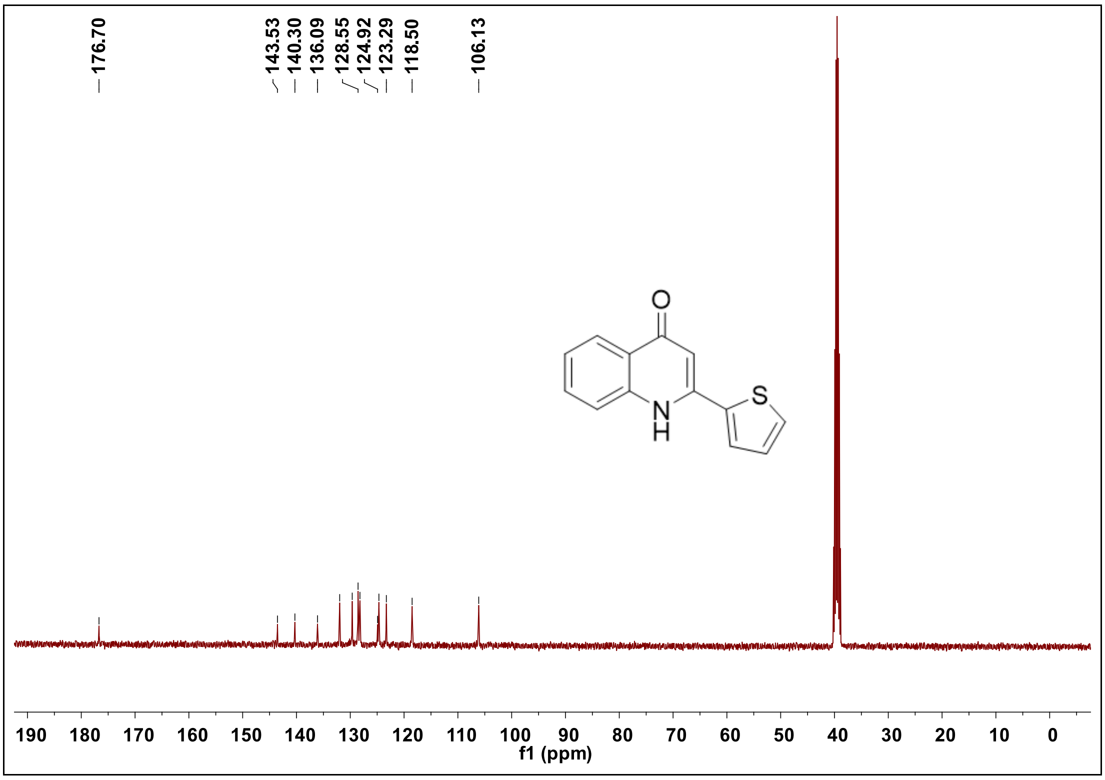

2-(pyridin-3-yl)quinolin-4(1H)-one (4i) ^{1}H NMR

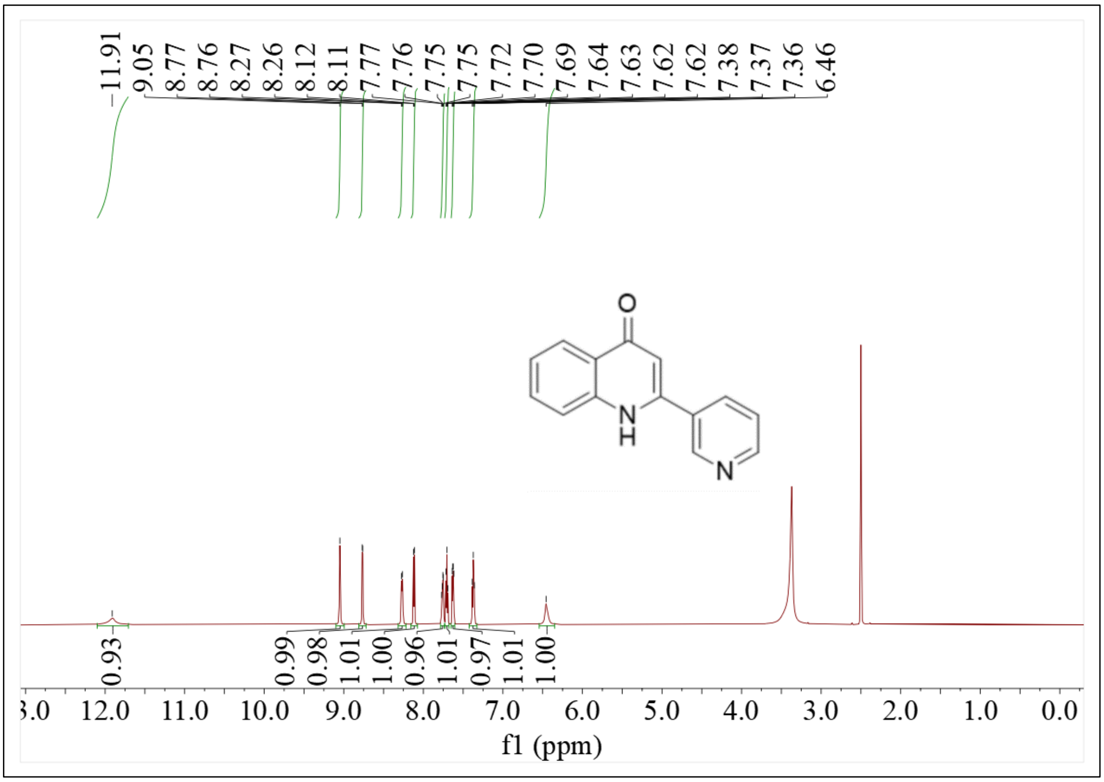

2-(pyridin-3-yl)quinolin-4(1H)-one (4i) ^{13}C NMR

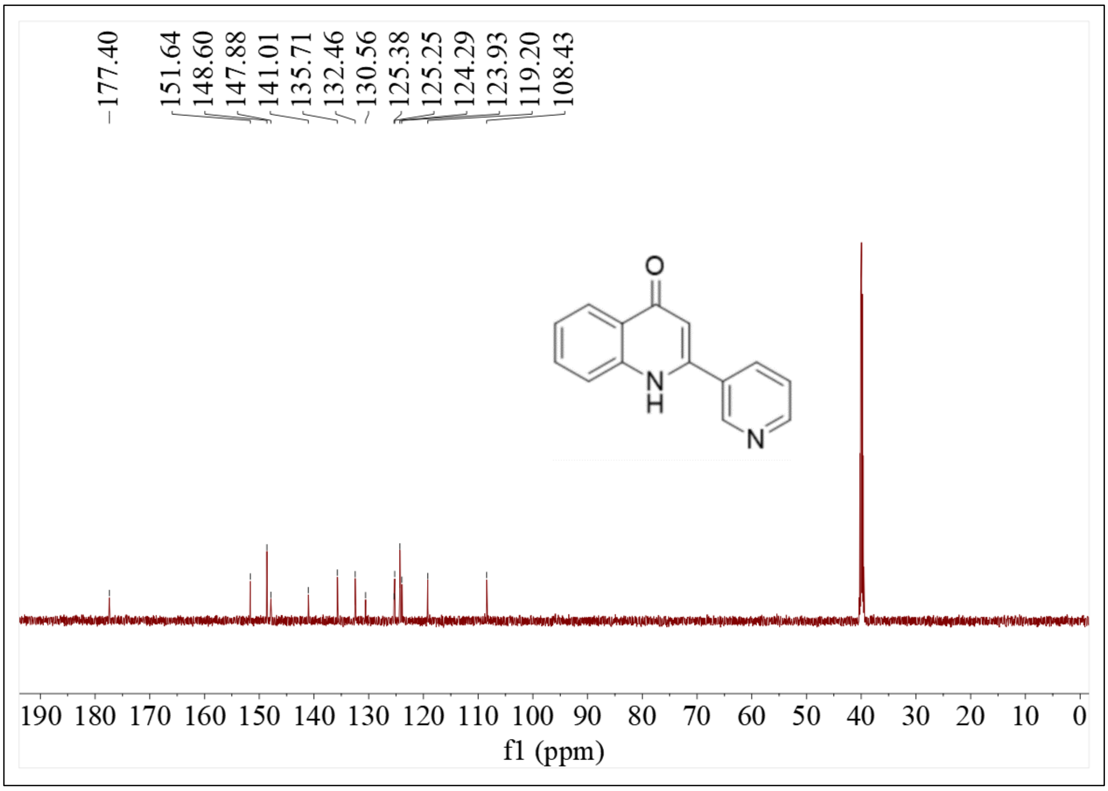

2-hexylquinolin-4(1H)-one (4j) ^{1}H NMR

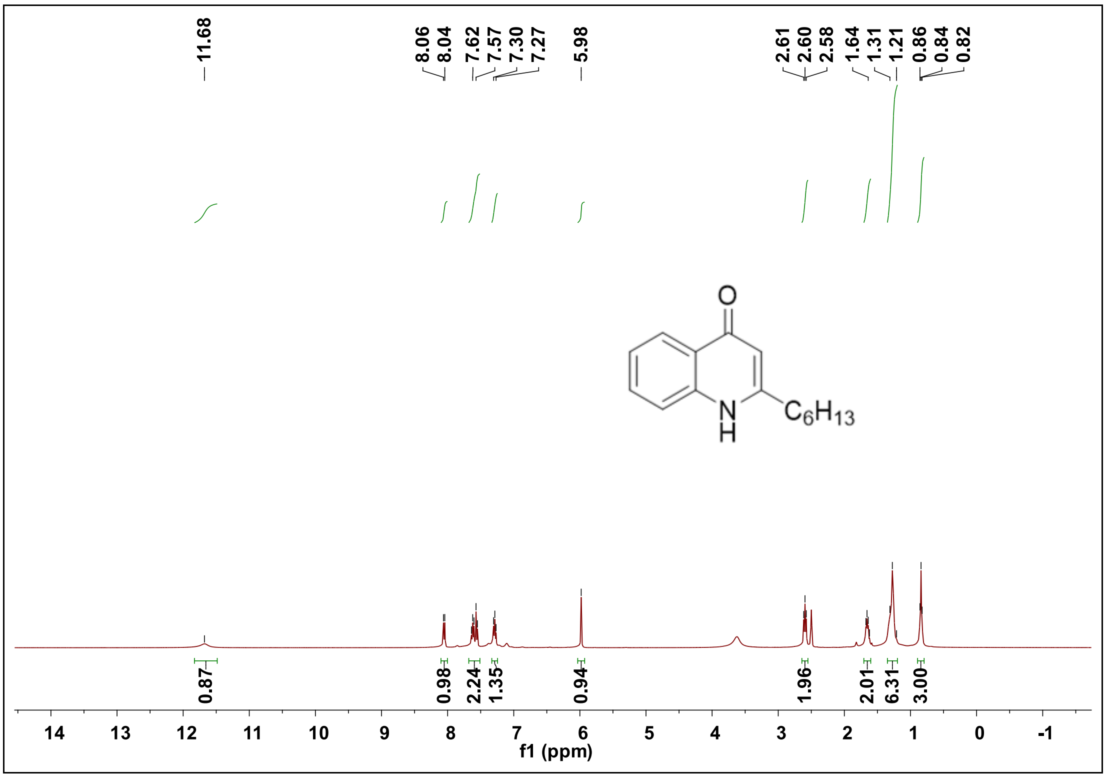

2-hexylquinolin-4(1H)-one (4j) ^{13}C NMR

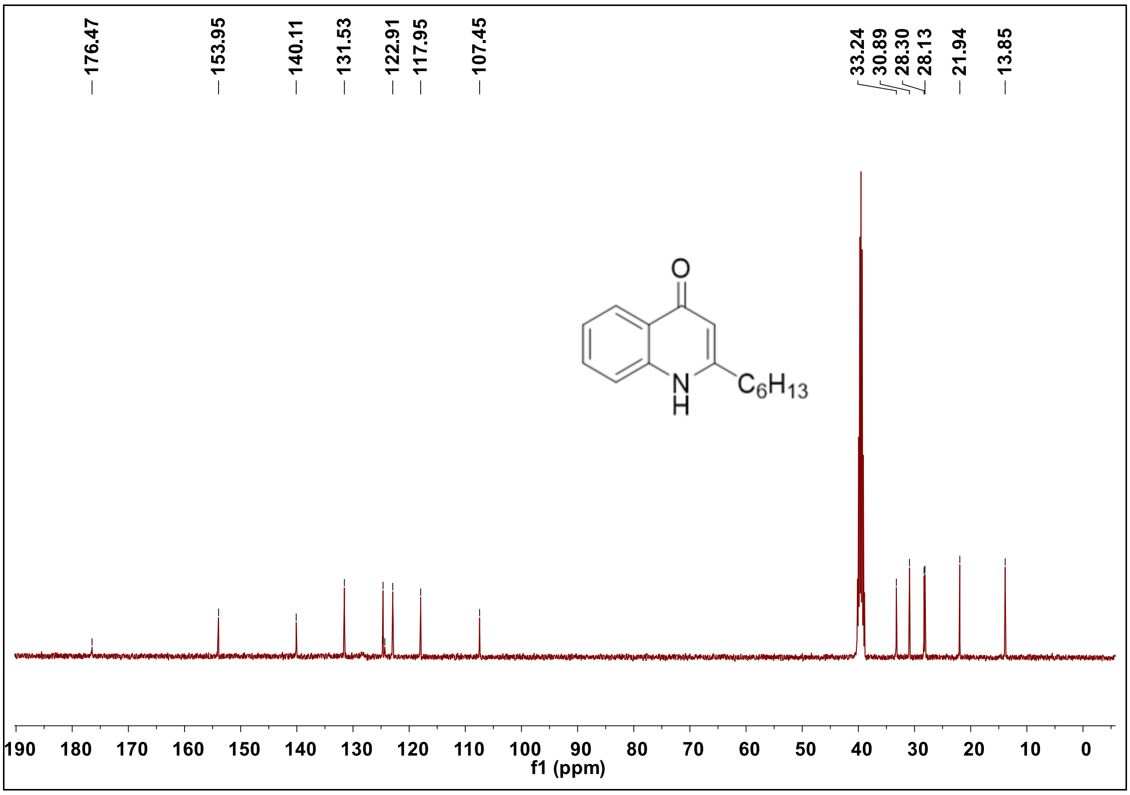

2-heptylquinolin-4(1H)-one (4k) ^{1}H NMR

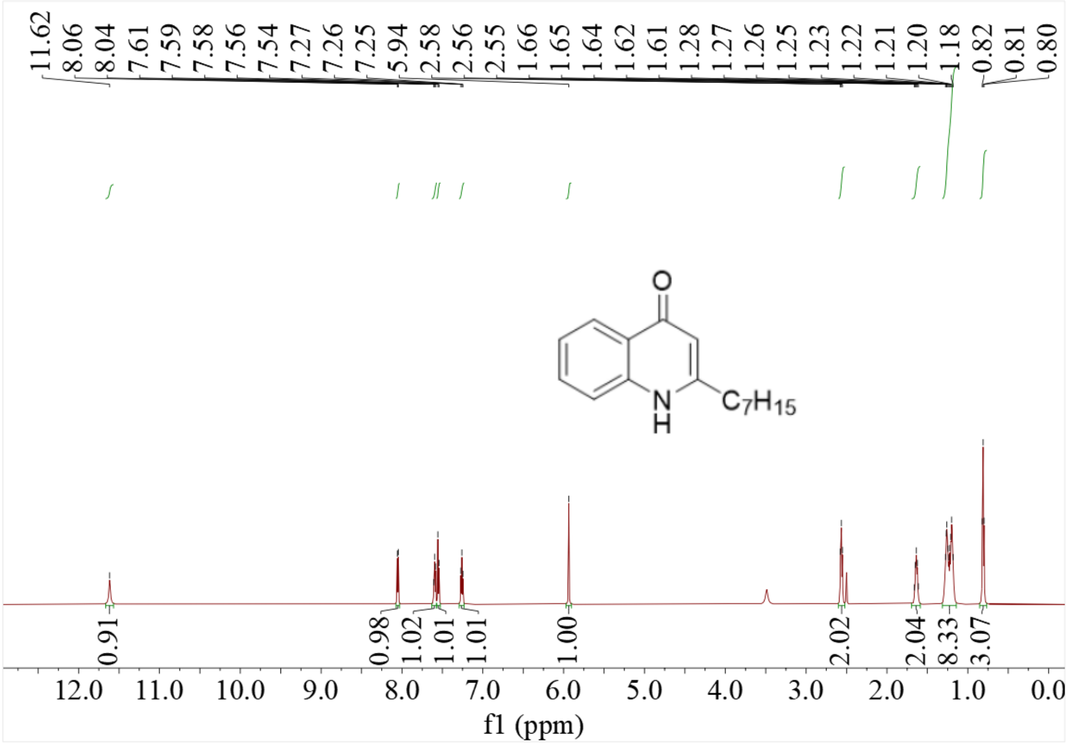

2-heptylquinolin-4(1H)-one (4k) ^{13}C NMR

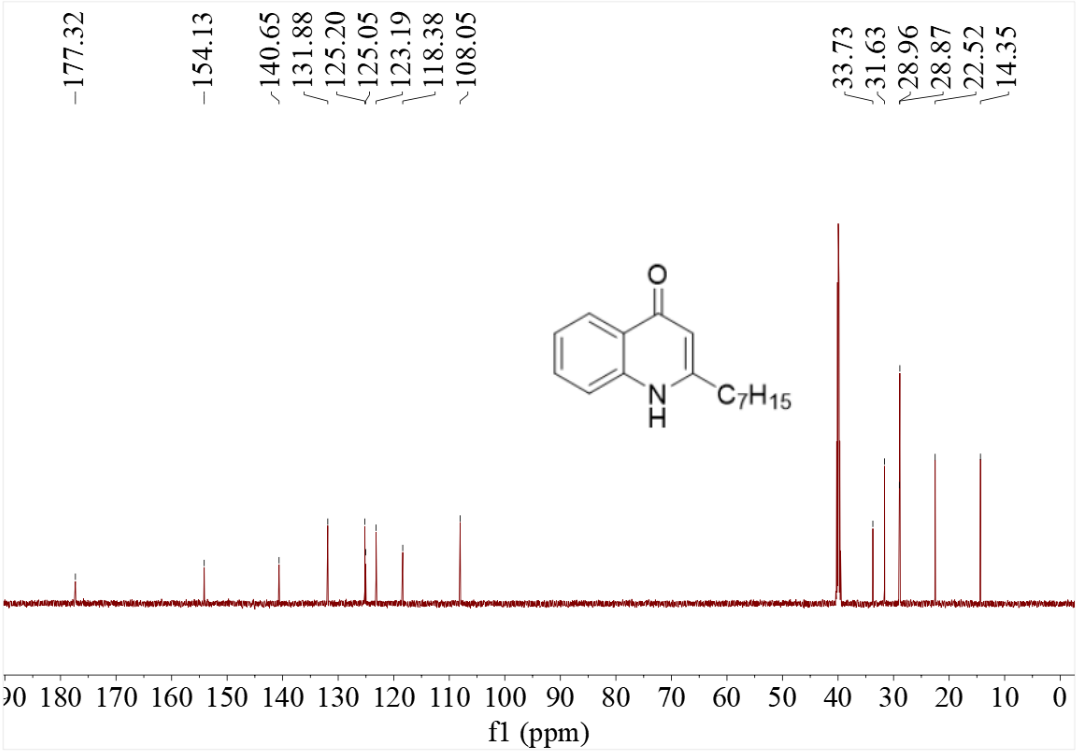

6-chloro-2-phenylquinolin-4(1H)-one (4l) ^{1}H NMR

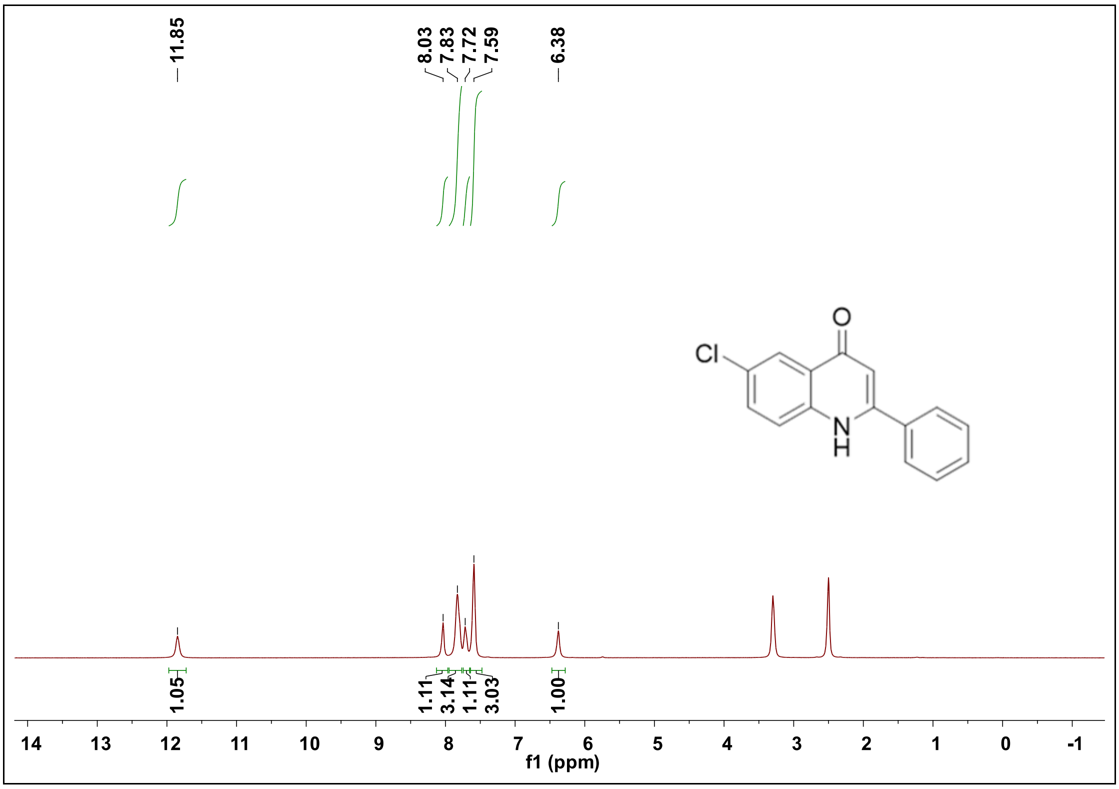

6-chloro-2-phenylquinolin-4(1H)-one (4l) ^{13}C NMR

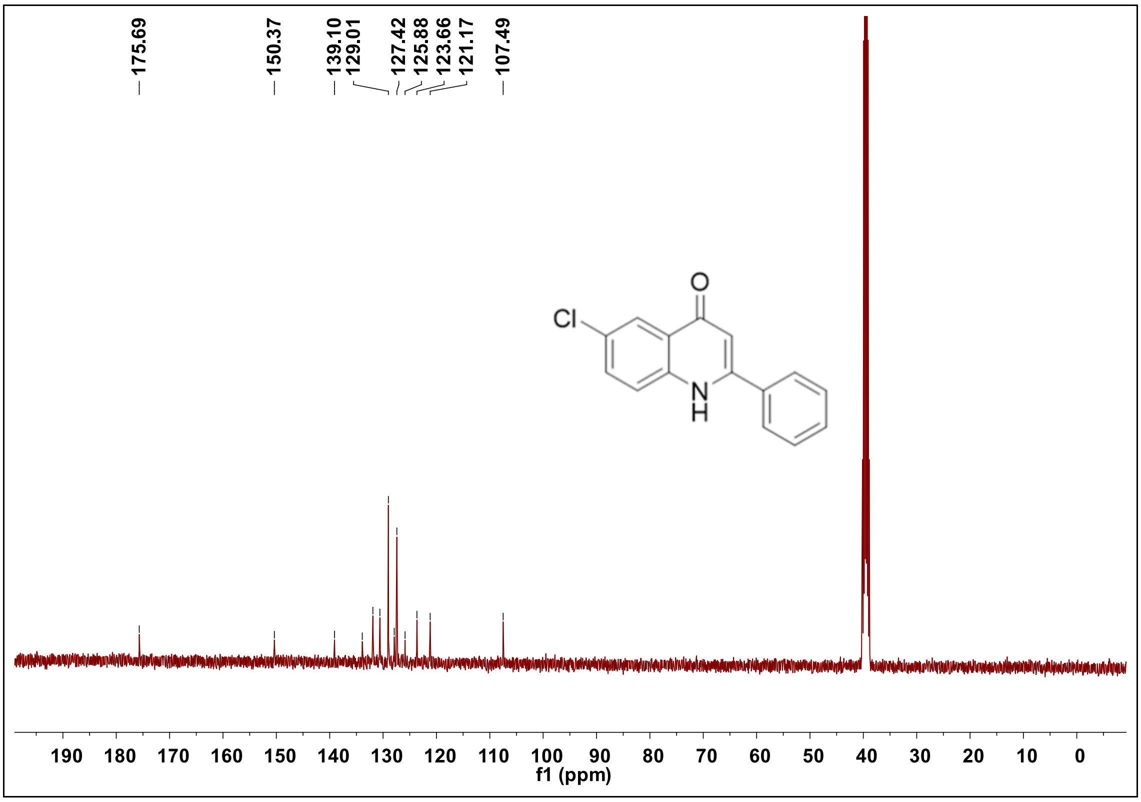

6-chloro-2-(4-methoxyphenyl) quinolin-4(1H)-one (4m) ^{1}H NMR

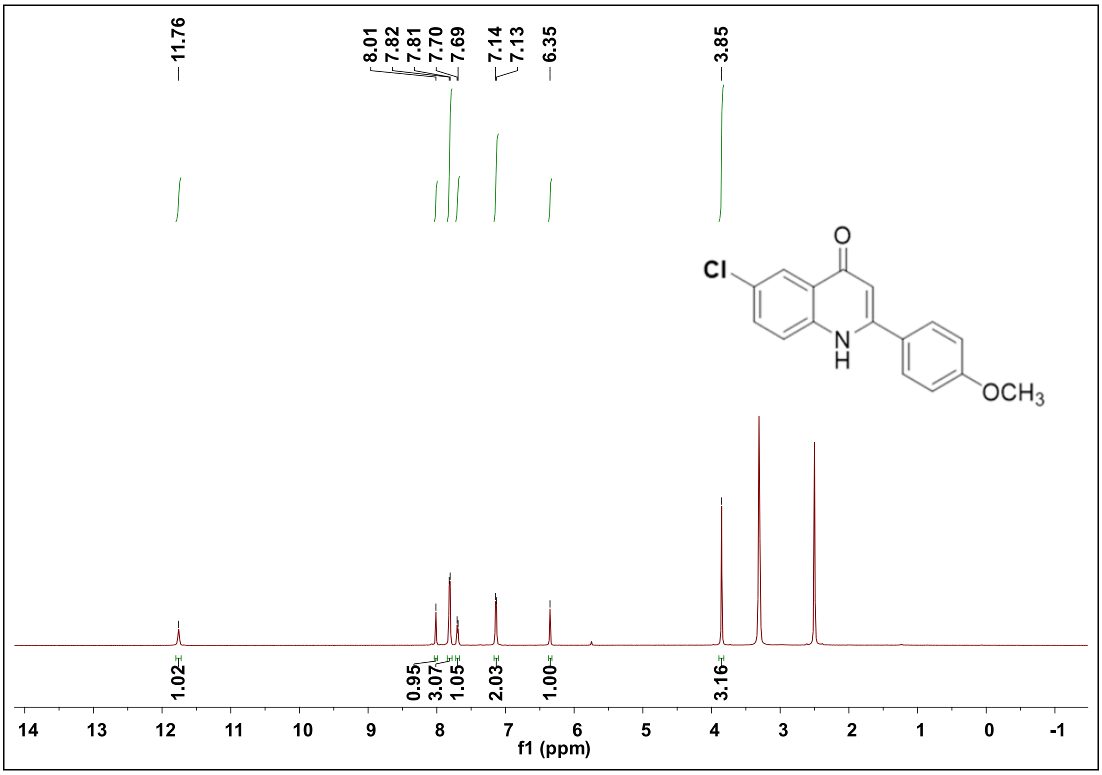

6-chloro-2-(4-methoxyphenyl) quinolin-4(1H)-one (4m) ^{13}C NMR

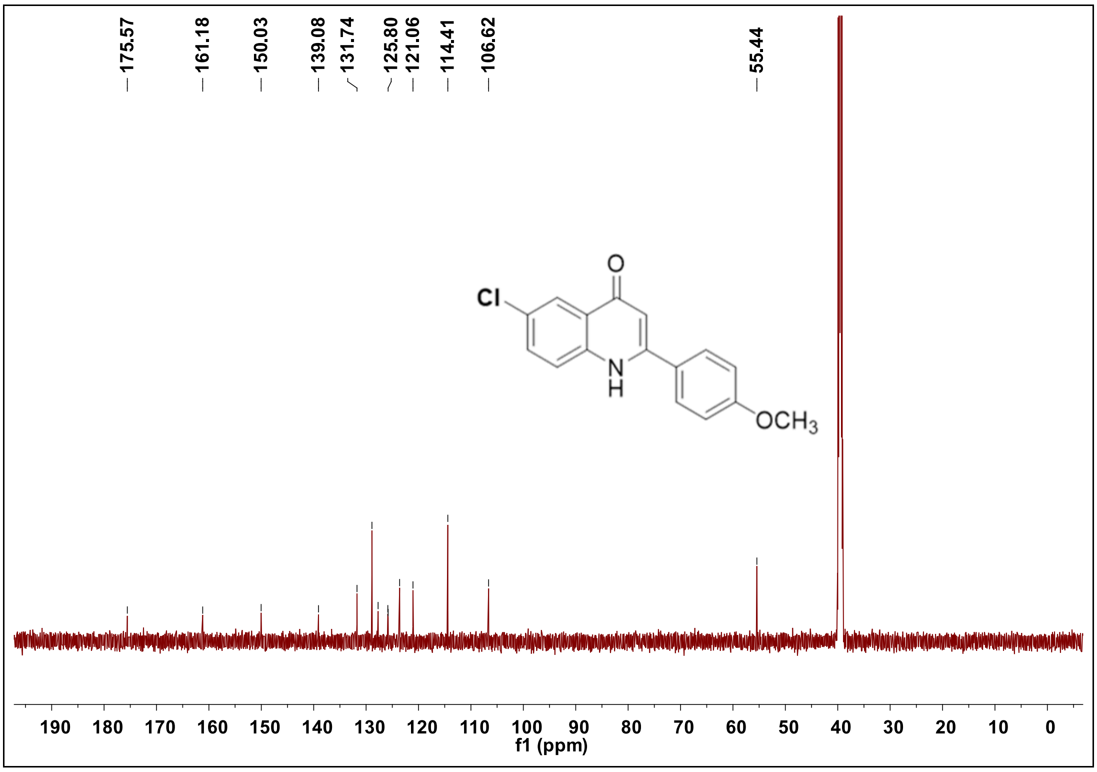

6-chloro-2-(p-tolyl) quinolin-4(1H)-one (4n) ^{1}H NMR

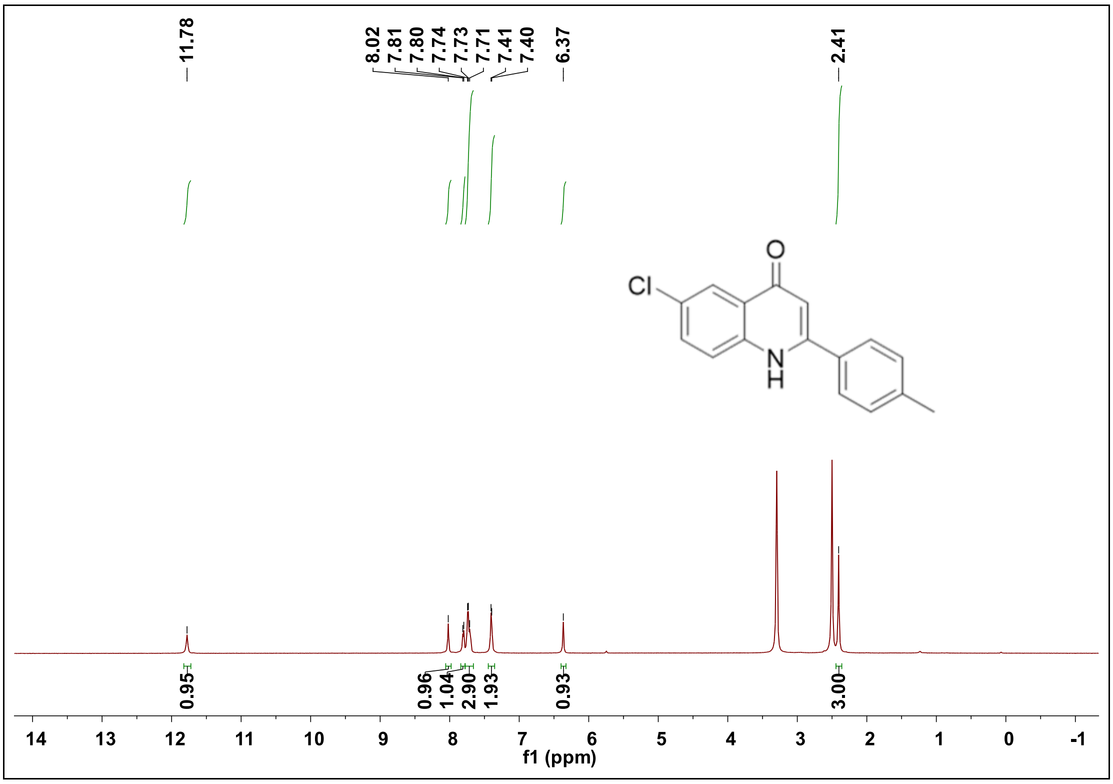

6-chloro-2-(p-tolyl) quinolin-4(1H)-one (4n) ^{13}C NMR

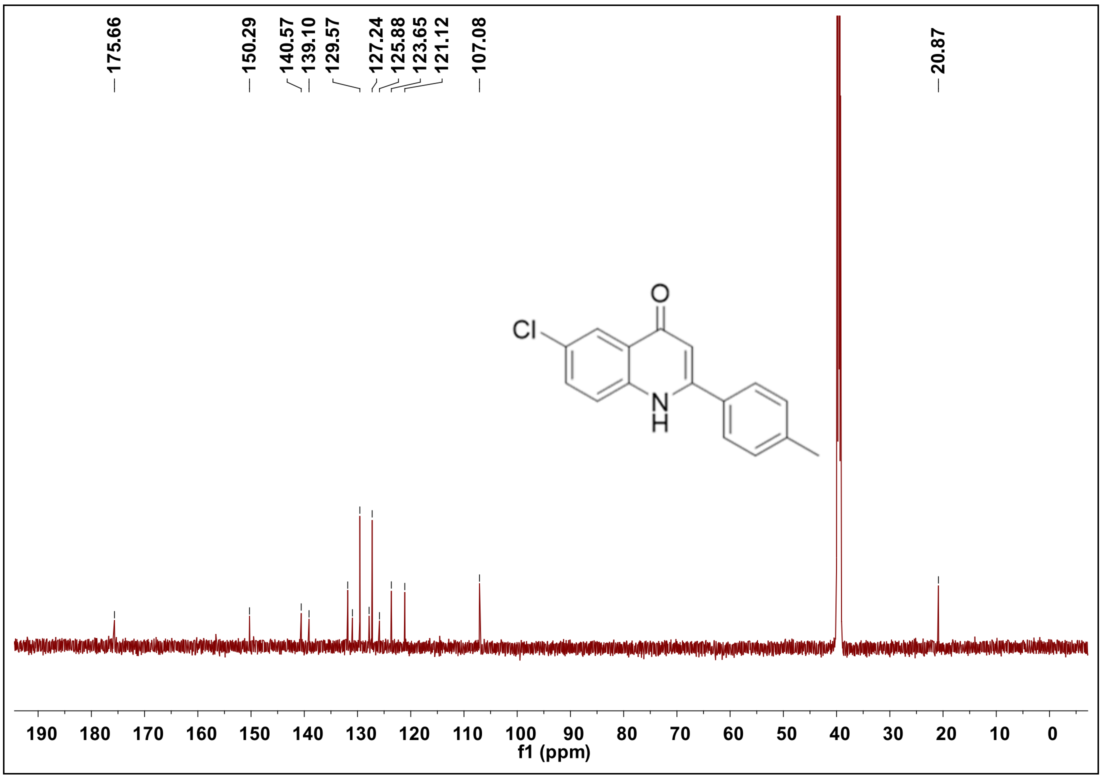

7-methyl-2-phenylquinolin-4(1H)-one (4o) ^{1}H NMR

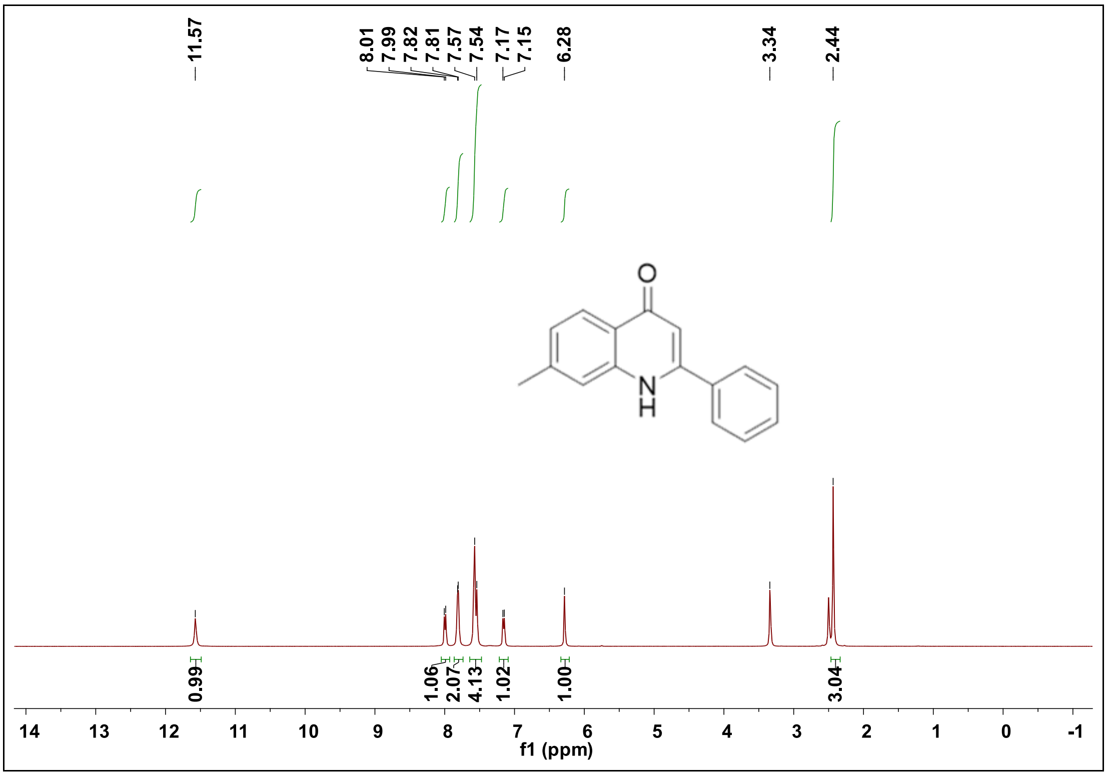

7-methyl-2-phenylquinolin-4(1H)-one (4o) ^{13}C NMR

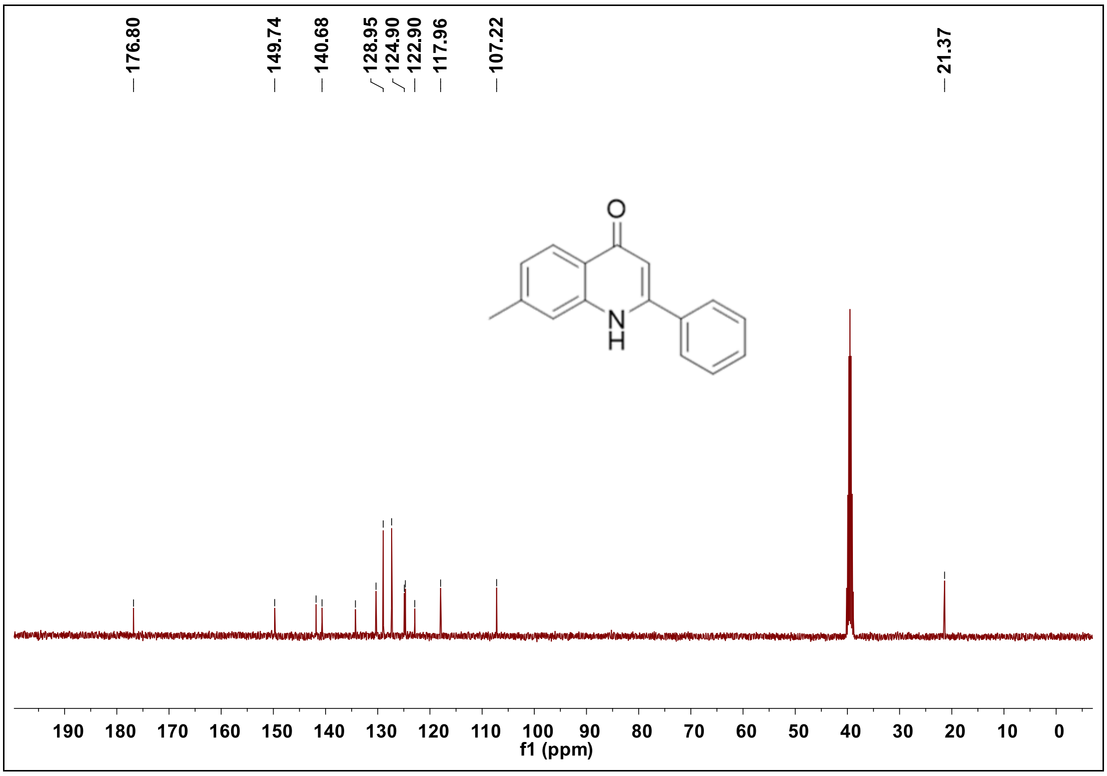

7-methyl-2-(m-methoxyphenyl) quinolin-4(1H)-one (4p) ^{1}H NMR

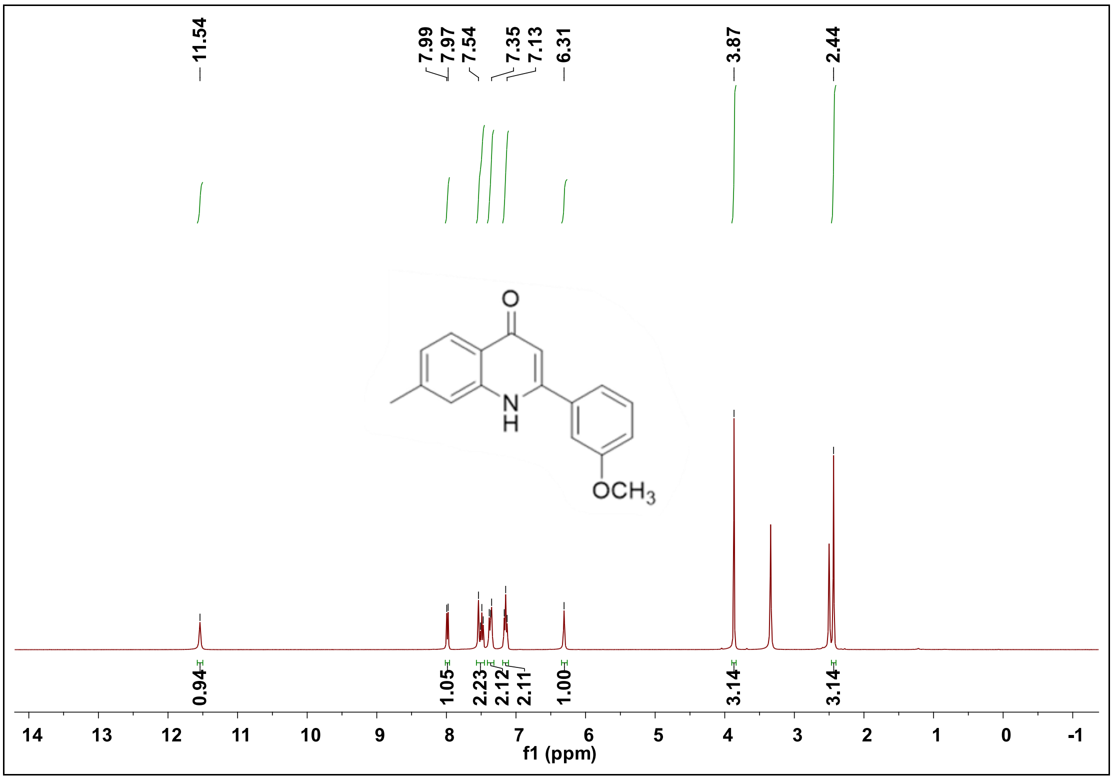

7-methyl-2-(m-methoxyphenyl) quinolin-4(1H)-one (4p) ^{13}C NMR

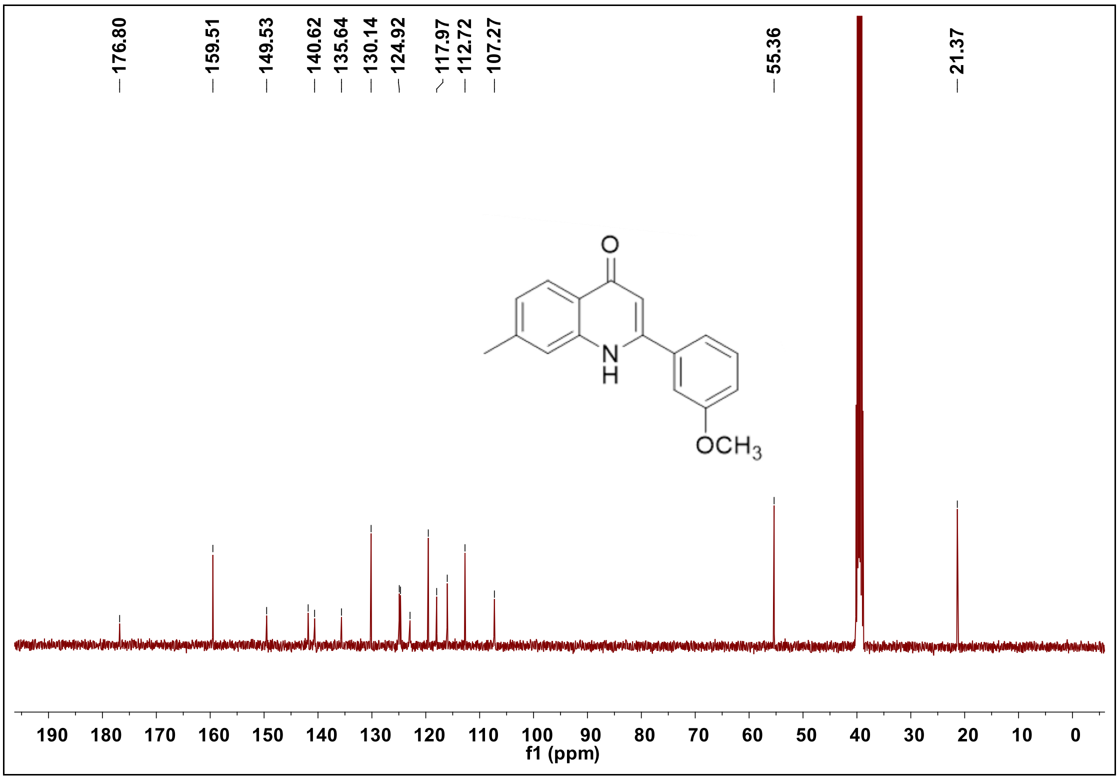

7-methyl-2-(p-methoxyphenyl) quinolin-4(1H)-one (4q) ^{1}H NMR

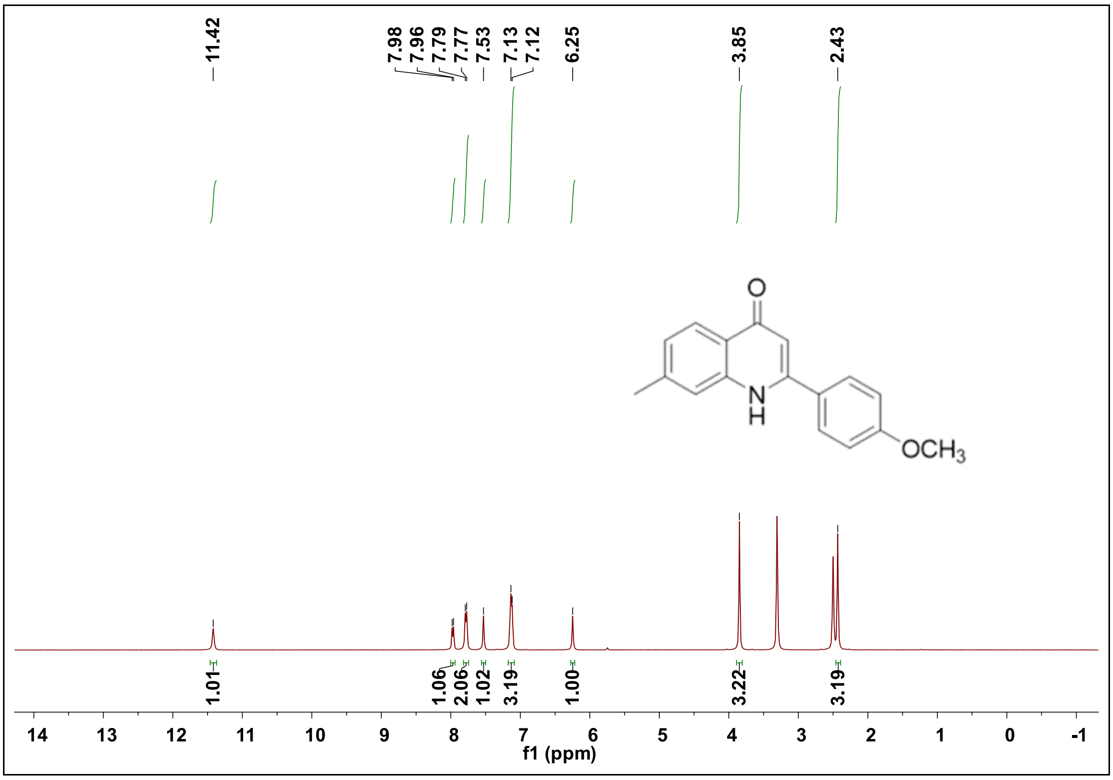

7-methyl-2-(p-methoxyphenyl) quinolin-4(1H)-one (4q) ^{13}C NMR

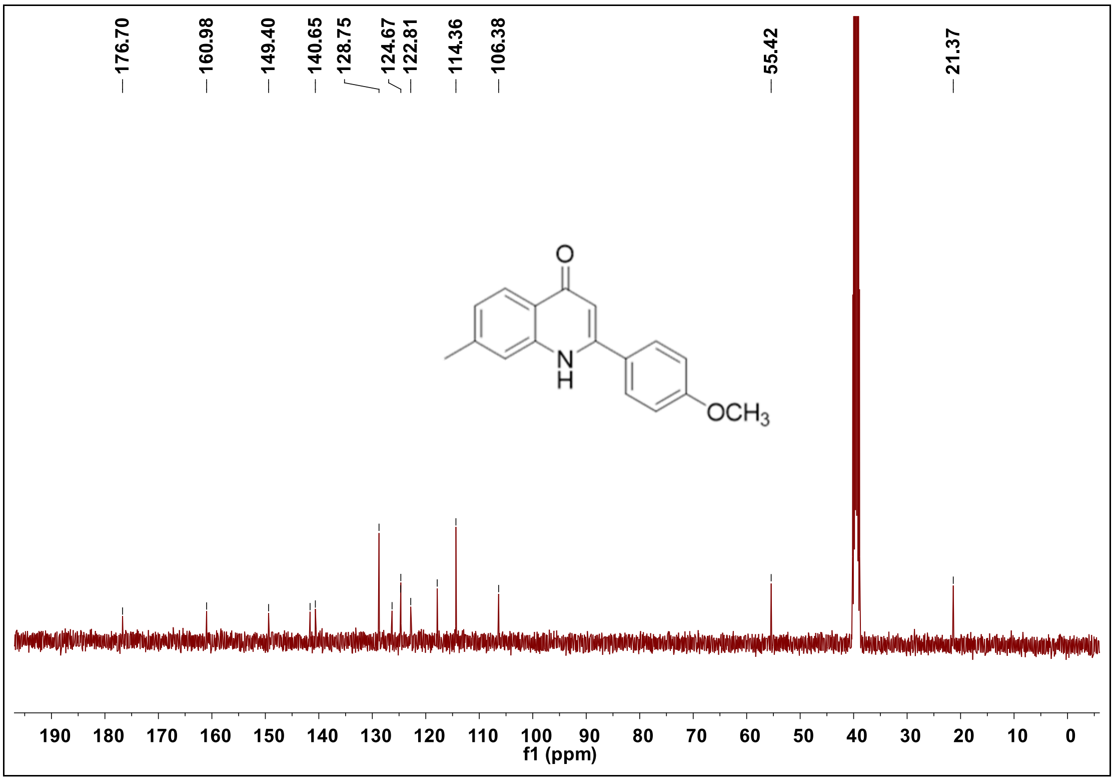

7-methyl-2-(m-tolyl) quinolin-4(1H)-one (4r) ^{1}H NMR

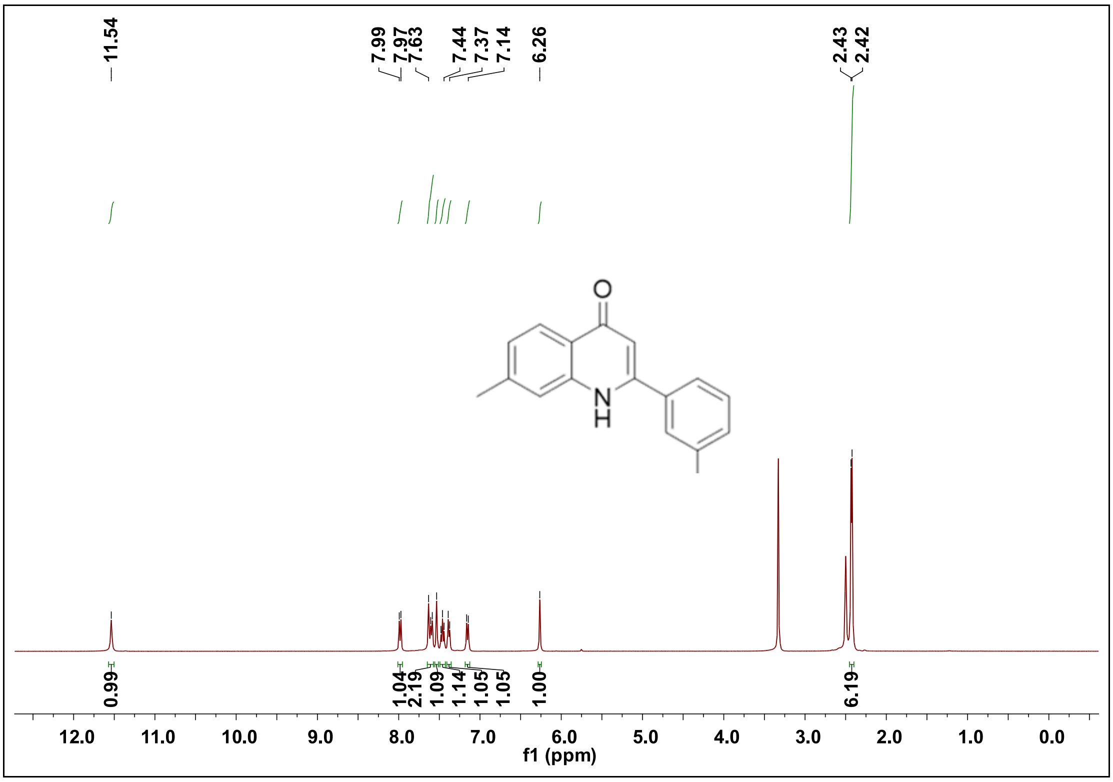

7-methyl-2-(m-tolyl) quinolin-4(1H)-one (4r) ^{13}C NMR

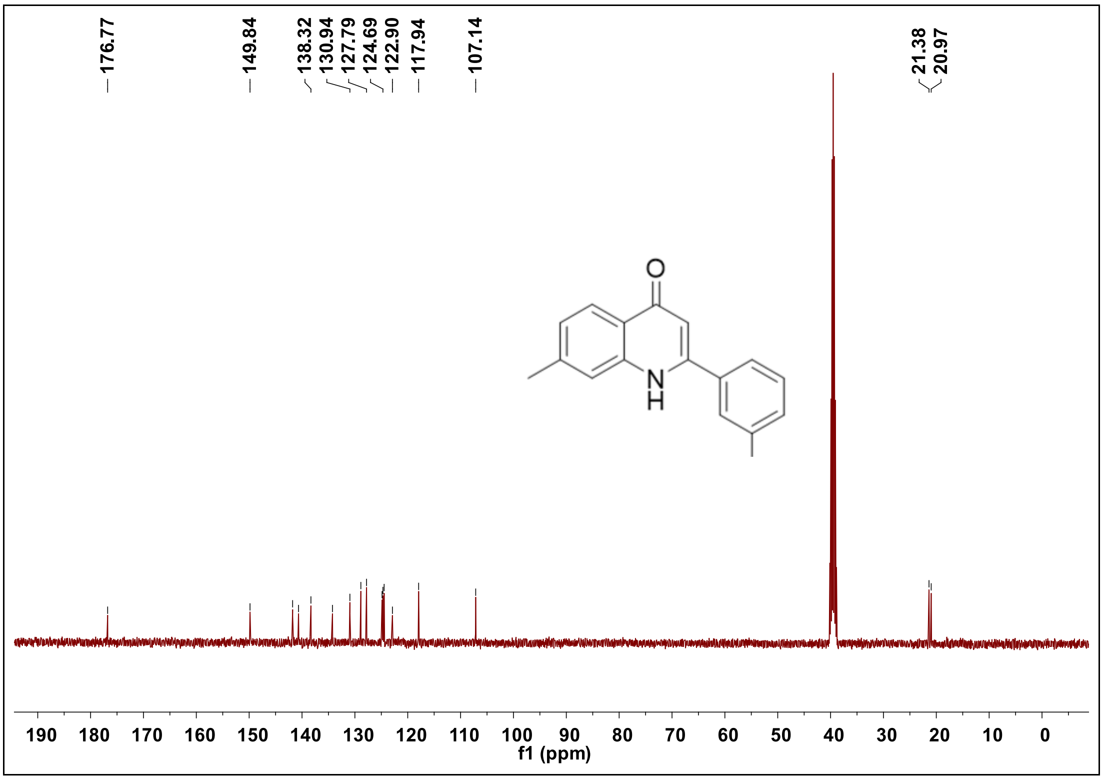

7-methyl-2-(p-tolyl) quinolin-4(1H)-one (4s) ^{1}H NMR

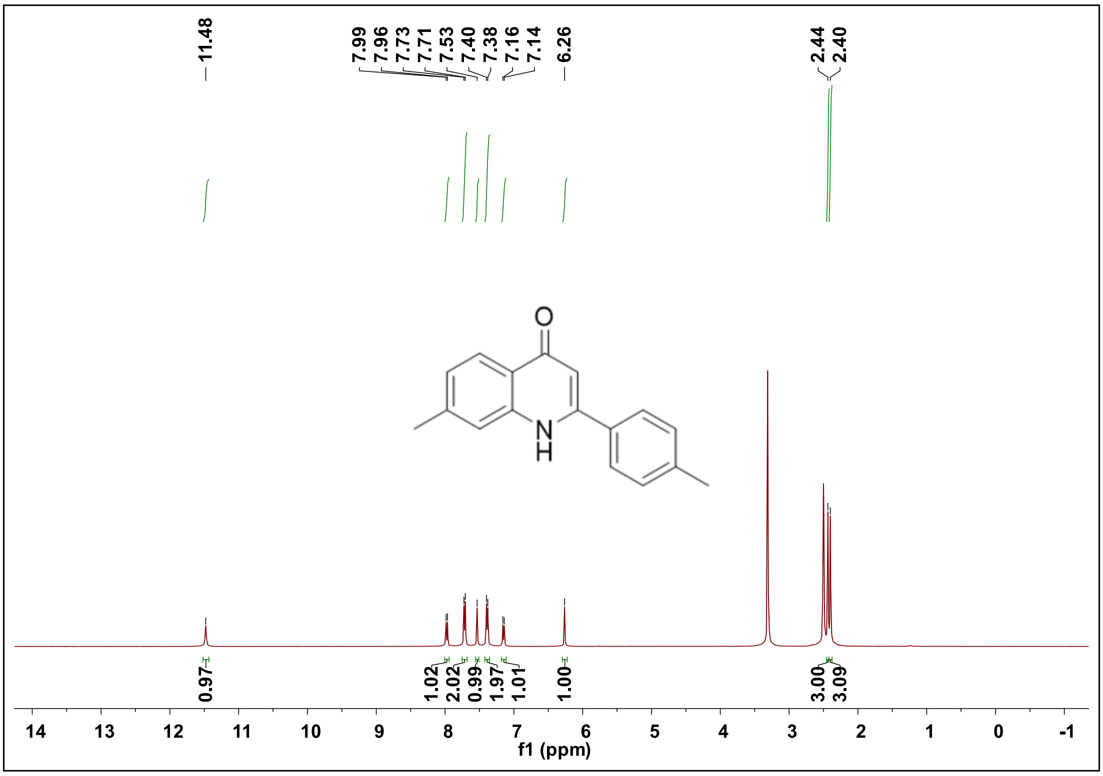

7-methyl-2-(p-tolyl) quinolin-4(1H)-one (4s) ^{13}C NMR

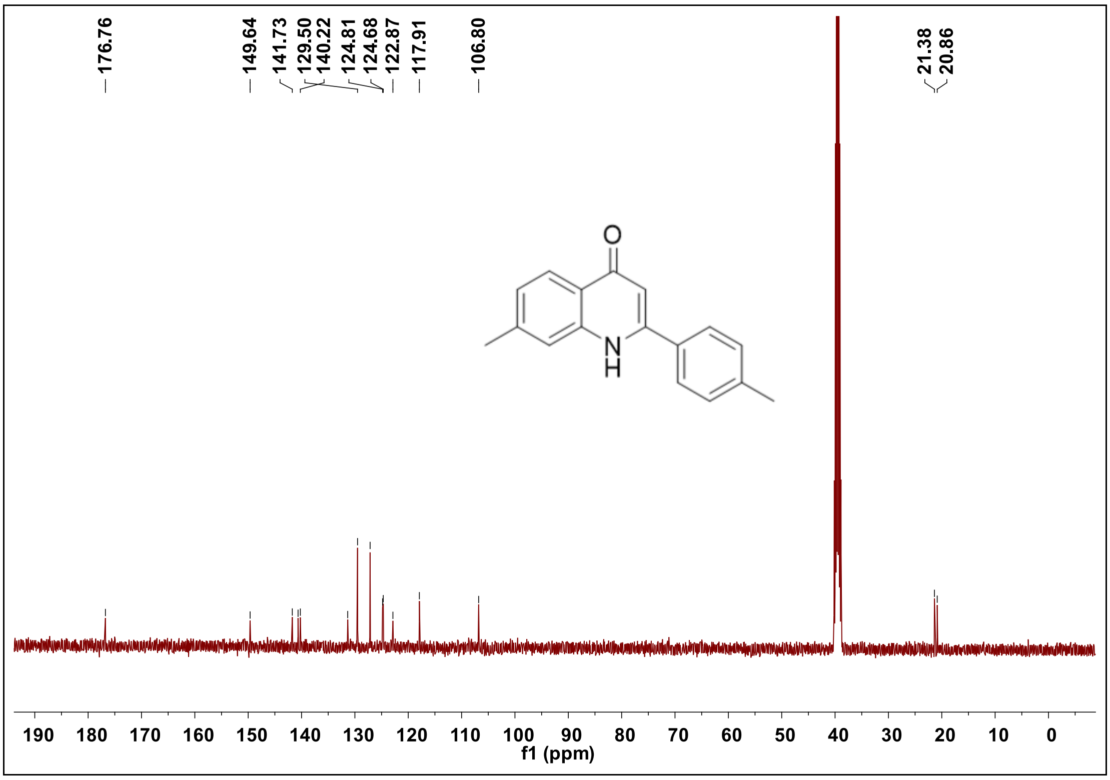

7-methyl-2-(4-butylphenyl) quinolin-4(1H)-one (4t) ^{1}H NMR

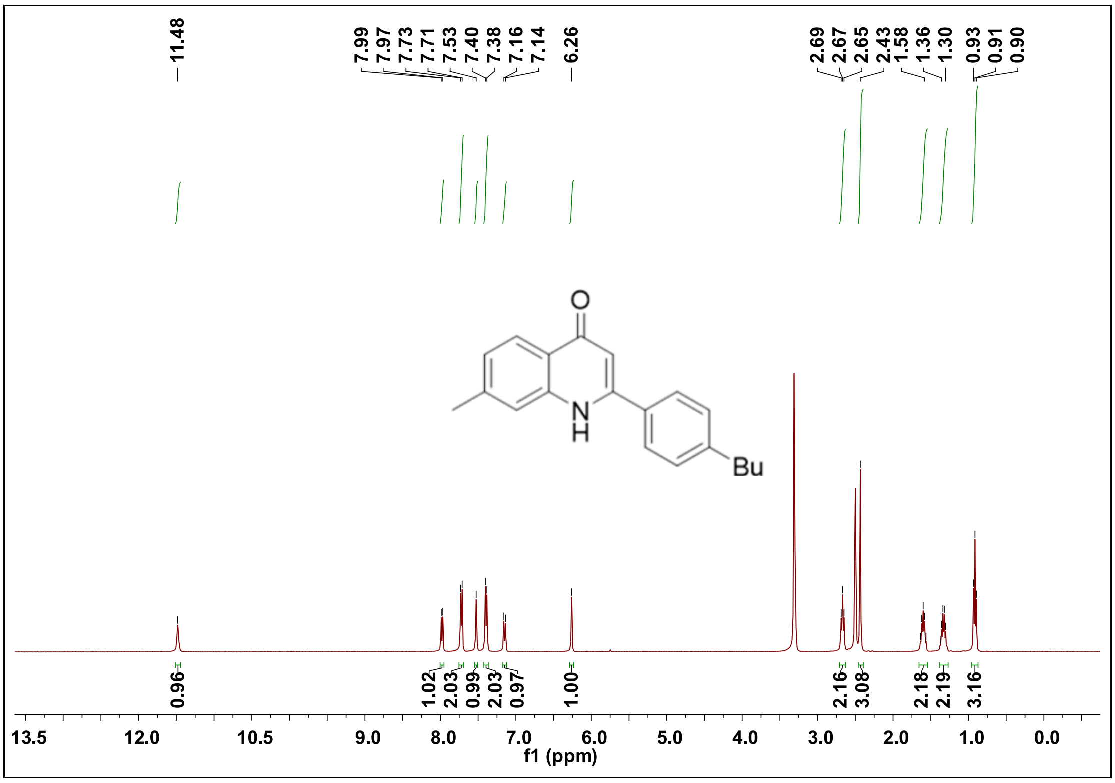

7-methyl-2-(4-butylphenyl) quinolin-4(1H)-one (4t) ^{13}C NMR

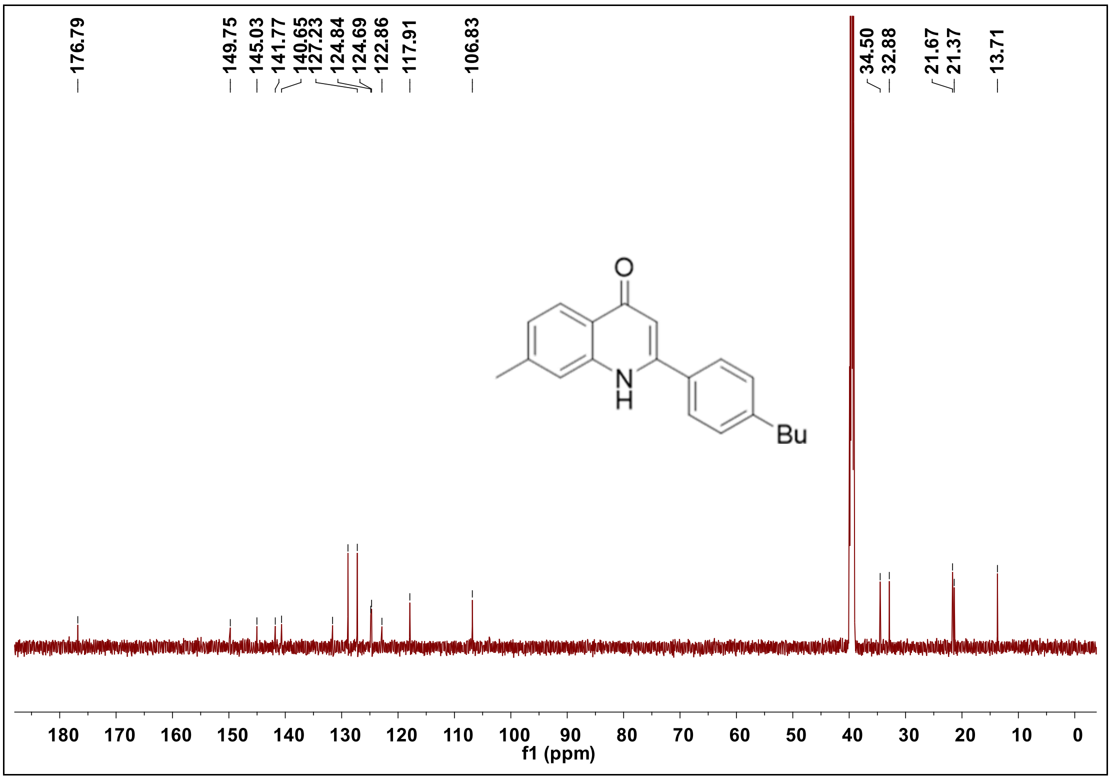

7-methyl-2-(4-(4-oxo-1,4-dihydroquinolin-2-yl)phenyl)acetonitrile (4u) ^{1}H NMR

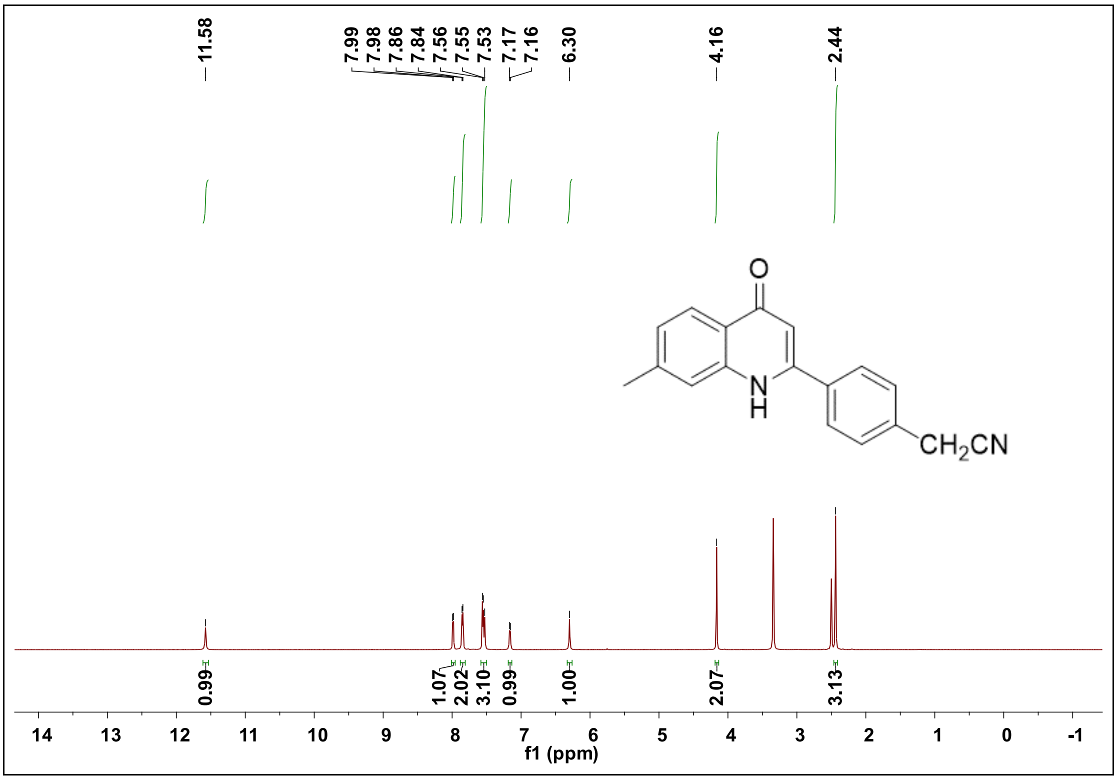

7-methyl-2-(4-(4-oxo-1,4-dihydroquinolin-2-yl)phenyl)acetonitrile (4u) ^{13}C NMR

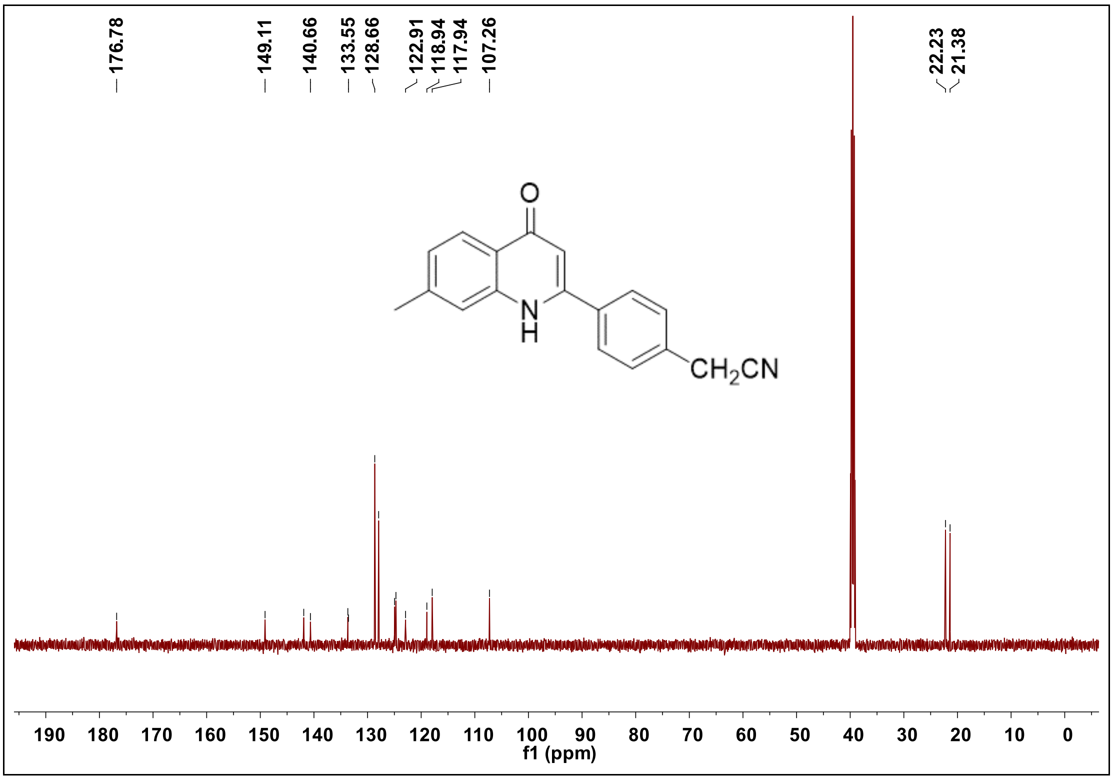

7-methyl-2-(thiophen-2-yl) quinolin-4(1H)-one (4v) ^{1}H NMR

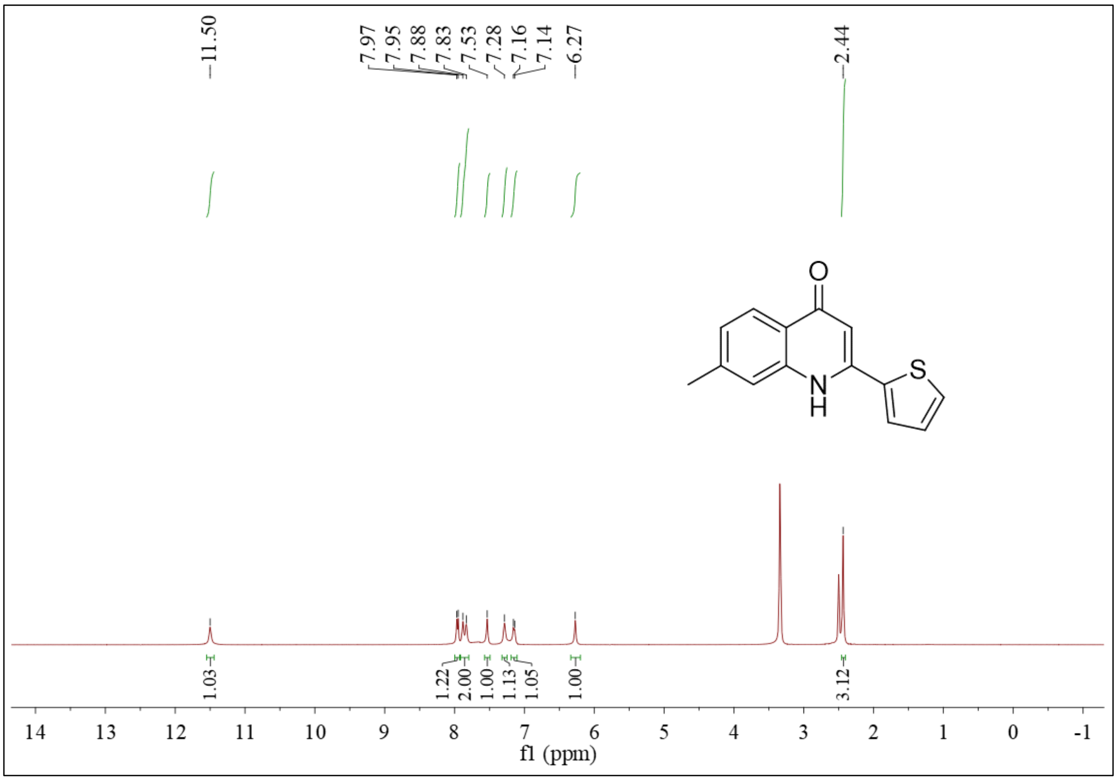

7-methyl-2-(thiophen-2-yl) quinolin-4(1H)-one (4v) ^{13}C NMR

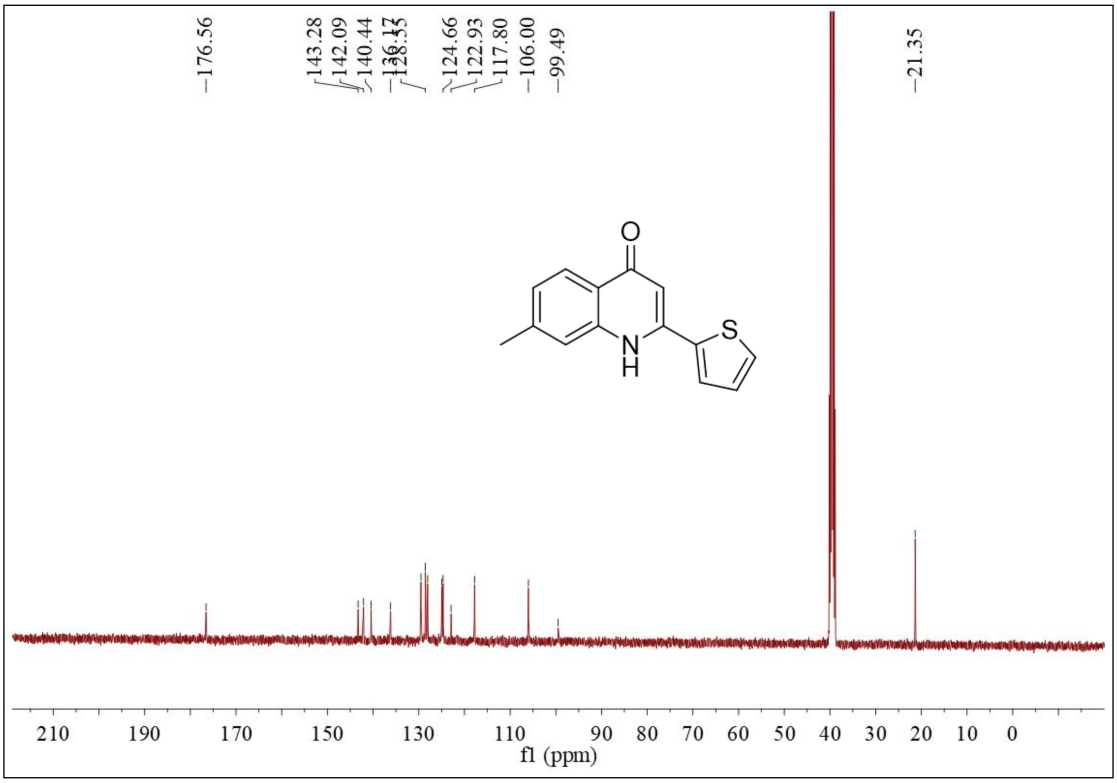

3. HRMS Spectra

Palladium complex **VI or VII [M+H]**+

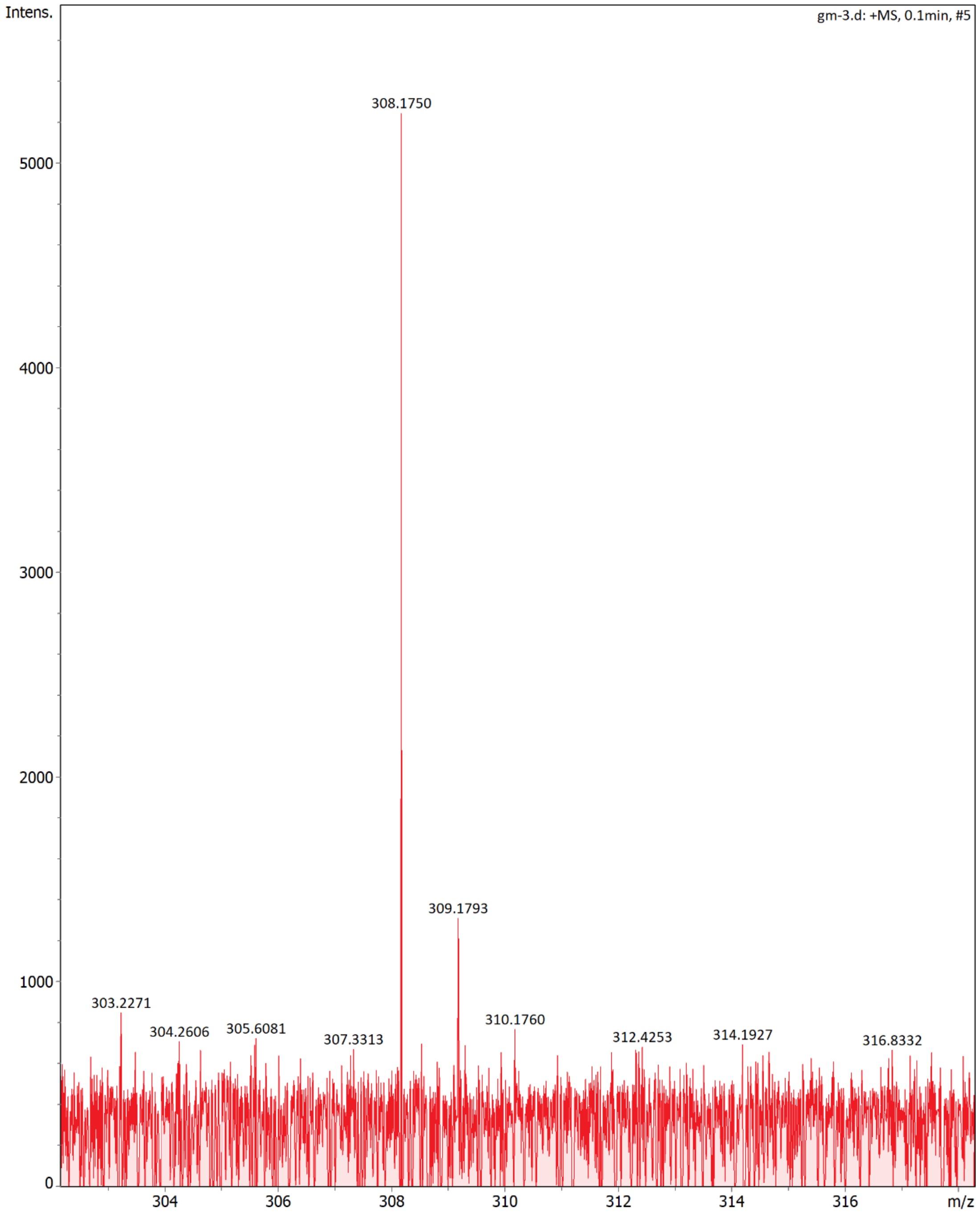

Supplement: Supplementary file 1 [file molecules-29-00850-s001.zip › molecules-2873738-supplementary.pdf]
